# Conservation of binding properties in protein models

**DOI:** 10.1016/j.csbj.2021.04.048

**Published:** 2021-04-25

**Authors:** Megan Egbert, Kathryn A. Porter, Usman Ghani, Sergei Kotelnikov, Thu Nguyen, Ryota Ashizawa, Dima Kozakov, Sandor Vajda

**Affiliations:** a Department of Biomedical Engineering, Boston University, Boston, MA 02215, United States; b Department of Applied Mathematics and Statistics, Stony Brook University, Stony Brook, NY 11794, United States; c Laufer Center for Physical and Quantitative Biology, Stony Brook University, Stony Brook, NY 11794, United States; d Department of Chemistry, Boston University, Boston, MA 02215, United States

**Keywords:** Structure prediction, Protein binding site, Protein–protein interaction, Quality measures, Binding hot spots, Protein mapping

## Abstract

We study the models submitted to round 12 of the Critical Assessment of protein Structure Prediction (CASP) experiment to assess how well the binding properties are conserved when the X-ray structures of the target proteins are replaced by their models. To explore small molecule binding we generate distributions of molecular probes – which are fragment-sized organic molecules of varying size, shape, and polarity – around the protein, and count the number of interactions between each residue and the probes, resulting in a vector of interactions we call a binding fingerprint. The similarity between two fingerprints, one for the X-ray structure and the other for a model of the protein, is determined by calculating the correlation coefficient between the two vectors. The resulting correlation coefficients are shown to correlate with global measures of accuracy established in CASP, and the relationship yields an accuracy threshold that has to be reached for meaningful binding surface conservation. The clusters formed by the probe molecules reliably predict binding hot spots and ligand binding sites in both X-ray structures and reasonably accurate models of the target, but ensembles of models may be needed for assessing the availability of proper binding pockets. We explored ligand docking to the few targets that had bound ligands in the X-ray structure. More targets were available to assess the ability of the models to reproduce protein–protein interactions by docking both the X-ray structures and models to their interaction partners in complexes. It was shown that this application is more difficult than finding small ligand binding sites, and the success rates heavily depend on the local structure in the potential interface. In particular, predicted conformations of flexible loops are frequently incorrect in otherwise highly accurate models, and may prevent predicting correct protein–protein interactions.

## Graphical abstract

**Figure:**
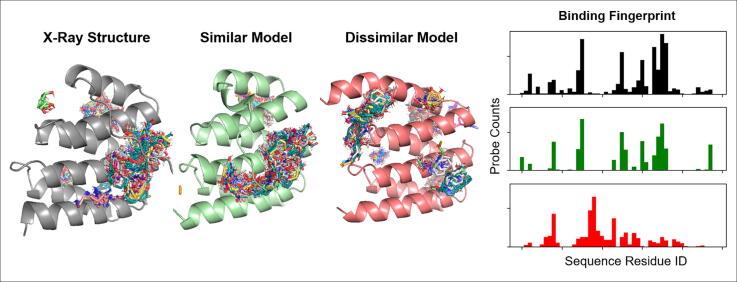

## 1 Introduction

Protein structure prediction is both a scientific challenge and an important application. The worldwide protein structure prediction experiment called Critical Assessment of protein Structure Prediction (CASP) has been providing an important platform for testing various methods and exchanging ideas [1], [2], [3], [4], [5], [6]. CASP is a large-scale community experiment, conducted every two years. The key feature is that participants make bona fide blind predictions of structures. Information about soon-to-be experimentally determined protein structures is collected and passed on to registered predictors [2]. During the past 26 years, CASP has monitored the state of the art in modeling protein structure from sequence. During this period, there has been substantial progress in template-based modeling of structure (using information from an evolutionarily related structural template), template-free modeling, and model refinement. Although CASP is essentially a competition, it has introduced a new area of reproducibility and openness in computational structural biology, and resulted an ever improving body of predictive methods.

The evaluators of CASP have developed a large variety of prediction quality measures [1], [2], [3], [4], [5], [6] (see also https://predictioncenter.org/casp12/doc/help.html). The most important ones are GDT_TS (GlobalDistanceTest_TotalScore), defined as GDT_TS = (GDT_P1 + GDT_P2 + GDT_P4 + GDT_P8)/4, where GDT_Pn denotes percent of residues under distance cutoff below nÅ, and GDT_HA (High accurate GDT), defined as GDT_HA =  (GDT_P0.5 + GDT_P1 + GDT_P2 + GDT_P4)/4. The global distance test (GDT) scores are effective for the automatic evaluation of predictions as they reflect absolute and relative accuracy of models for a wide range of target difficulty. These measures focus on the accuracy of the backbone atoms, but similar measures have been defined for side chain atoms as GDC_SC (Global Distance Calculation for sidechains), which uses a characteristic atom near the end of each sidechain type (instead of CA's) for the evaluation of residue-residue distance. A similar measure, GDC_ALL, takes into account the protein all non-hydrogen atoms. The ranking of models is determined by the CASP organizers using these measures.

One important problem the organizers of CASP have been struggling with is to better define whether and in what types of applications will protein structure prediction be useful, or more generally, to better describe the biological and functional relevance of CASP predictions [7], [8], [9]. This problem is far from simple, since proteins can act as enzymes, cell surface receptors, transporters, ion channels, agents in the immune system, and drug targets [10], [11]. They can also participate in genetic and metabolic regulation and in signal transduction [12]. Information on structure has varying importance in these applications, as the transition from structure to function is generally not straightforward. In the previous CASP rounds, the organizers sought to emphasize the biological implications of structure prediction by asking the authors providing the experimental structures to describe their rationale for pursuing structure determination [7], [8], [9]. Such information was given for some targets, and ranged from interest in the oligomeric structure of the protein, the positions of ligands in the experimental structure, and structural explanations of missense mutations associated with disease [7]. However, the small number of responses from the depositors limited the generality of the analysis.

In the recent CASP rounds, efforts have been devoted to exploring whether the models can replace the X-ray structures in predicting protein properties, primarily interactions with other molecules. Dunbrack and coworkers [7] assessed the utility of the CASP11 results to perform quantifiable tasks related to biological function, including (i) protein–protein docking, (ii) drug design (small ligand docking), (iii) assessing the missense mutations. The part of their study regarding protein–protein docking employed the ClusPro program [13], while their main goal was to measure the Jaccard similarity of contacts (Q score) in the ClusPro-modeled homodimer with those in the experimental structure [7]. Regardless of the measure of model accuracy (GDT_TS, RMSD, LDDT) most of the models were not able to form the correct homodimer in any of the top clusters produced by ClusPro. They also tested whether the protein-small molecule docking programs SwissDock [14] and Autodock Vina [15] were able to re-create a nitroreductase-flavodoxin complex using the nitroreductase models submitted by the CASP participants. For CASP12 the function evaluator team examined nine sites with known ligand binding and nine sites that were expected or were suggested by experimental authors for small molecule binding [8]. The sites in the models and the X-ray structures of the targets were compared in terms of their microenvironments, defined by a variety of physicochemical properties collected over concentric spherical shells around selected functional centers. It was found that the overall structural quality correlated with functional utility, but the best-ranked predictions generally did not have the best functional quality. The team also analyzed features from protein assemblies of two targets that had active sites in the protein–protein interface. Although focusing on binding rather than any property made these studies better defined, binding site information was available only for a few targets, and in CASP13 the investigators returned to the original idea of analyzing functional and biological significance based on the descriptions provided by the authors of the structures [9].

One of the unifying features of a protein function is the ability to interact with other molecules in the cell, including small ligands, proteins, DNA, or RNA. Thus, in this paper we focus on molecular interactions and study essentially all targets of CASP12 to determine how well the models retain the surface properties that are important for binding, either to small molecule ligands or to other proteins. The motivation for focusing on CASP12 is that X-ray structures have already been deposited in the Protein Data Bank, and papers related to the structures have also been published for many targets, which enables better understanding of the underlying biology. We hope that our results will provide examples of potential quality measures relevant to molecular interactions, as such are not currently available in CASP. To explore the small ligand binding properties of the target proteins and their models we use a computational method based on protein mapping, also known as solvent mapping. The method has been introduced by Ringe and coworkers, who determined structures of target proteins in aqueous solutions of several organic solvents by X-ray crystallography [16], [17]. Superimposing the resulting structures demonstrated that the number of interactions between a residue and the probes predict the importance of the residue for ligand binding. In particular, the small organic “probe” molecules were found to cluster at binding hot spots that provide major contributions to the free energy of ligand binding [18], [19], [20]. The clustering of small organic molecules at ligand binding sites can also be observed by NMR, as demonstrated by Fesik and colleagues [21]. They showed that the fragments cluster at ligand binding sites, and described such regions as “hot spots on protein surfaces” [22]. The X-ray and NMR based experiments formed the basis for fragment based ligand design, an increasingly successful approach to drug discovery [23], [24].

The potential of X-ray structures and models of the CASP12 targets to bind small molecules will be explored using the FTMap program, which was developed as a computational analogue of the above protein mapping experiments [25], [26]. We generate distributions of molecular probes around the target protein, and count the number of interactions between each residue and the probes, resulting in a vector of interactions we will call the binding fingerprint. The similarity between two fingerprints, one for the X-ray structures and the other for a model of the protein, will be determined by calculating the correlation coefficient between the two vectors. As will be described, we studied models for 51 regular targets, as well as models for 31 refinement targets, the latter were selected for exploring methods designed to achieve higher prediction accuracy. Based on the increased scale of the analysis we were able to investigate the relationships between the conservation of the binding surface and classical measures of model quality such as GDT_TS. In addition to the global characterization of the protein surface in terms of the probe-residue interactions we studied the clustering of probes at specific sites, since such clusters provide information on binding hot spots that can be related to specific ligand binding. It will be shown that the clusters formed by the probe molecules reliably predict binding hot spots and ligand binding sites in both X-ray structures and the models with reasonable accuracy, but ensembles of models may be needed for assessing the availability of proper binding pockets. Unfortunately binding site information was available only for nine CASP12 targets, considered the “holo” structures by Liu et al. [8], and hence the global analysis based on the FTMap fingerprints is potentially more important, since it can be applied to many targets.

The second property of interest is the ability of the models to reproduce the protein–protein interactions observed in the X-ray structures. As mentioned, Dunbrack and co-workers considered this problem for a number of CASP11 targets [7]. Two factors enabled us to make this approach somewhat more informative when we apply it to the CASP12 targets. First, we have 23 regular and 14 refinement targets that are subunits of multimeric structures, and in all cases the X-ray structures of the complexes have been deposited to the PDB. Thus, in addition to docking models to the X-ray structure of the partner protein, we can also dock the subunits extracted from the complex, and thus to determine whether or not using models leads to a substantial drop in prediction quality. Second, we used the accuracy measures established by the CAPRI (Critical Assessment of Predicted Interactions) experiment [27], [28] and the widely used quality evaluation program DockQ [29], which enabled us to determine the numbers of acceptable, medium, and high accuracy docked structures. Using these measures, we were able to compare our results to the usual success rates observed in docking separately crystallized protein structures.

## 2 Methods

### 2.1 Selection of regular targets for the analysis of surface binding properties

The CASP12 competition was comprised of 77 regular targets. However, for the analysis of the small molecule binding we considered only the regular targets that had experimentally determined structures deposited in the Protein Data Bank (PDB) by March 2020, resulting in 51 targets (Table 1SI). The structure for each of these 51 targets was identified by a BLAST search of the sequence, provided on the CASP12 website, against the PDB. We note that wherever possible we selected PDB structures that matched the PDB code provided on the CASP12 website. Thus, a total of 26 regular CASP12 targets were removed from the analysis for either having no experimental structure (T0867, T0871, T0874, T0875, T0876, T0881, T0888, T0890, T0896, T0897, T0898, T0899, T0901, T0905, T0906, T0913, T0923, T0934, T0941, T0946, T0947) or being duplicates of previous targets (T0929, T0930, T0931, T0932, T0933). Table 1SI also shows the query cover and percent identity, both determined by BLAST, for each target sequence. With four exceptions the sequence of the structure in the PDB covers at least 97% of the CASP sequence, and the percent identity exceeds 96%. The experimental structures were frequently longer, and were trimmed (if needed) to match the sequence of the target or target domain.

The CASP12 assessment team ranked the submitted models according to their GDT_TS. These rankings, along with other individual score metrics are published on the CASP12 website (https://predictioncenter.org/casp12/index.cgi) for each target. For the analysis described in this paper, we considered only the models ranked 1–5 by GDT_TS, and refer to them as the Top 1, Top 2, etc. models throughout the paper. In the case of a tie (i.e. two or more models with the same rank in the top 5), we considered the top 5 models listed in the CASP12 results file. For example, T0863 has an 11-way tie for rank 4, so the top 5 models for T0863 are taken as Rank 1, 2, 3 and the first two targets listed as rank 4. In addition, if duplicate models existed in the top 5, defined by having equivalent GDT_TS, GDT_HA and GDC_SC to two significant digits, the duplicate was removed and subsequently ranked models were added to comprise the top 5 unique models for the target.

### 2.2 Selection of refinement targets for the analysis of surface binding properties

The organizer of CASP selected 42 refinement targets to test whether the quality of initial models can be further improved. The goal was to select interesting targets but discard cases where the experimental structure is dictated by extensive multimeric interactions, or where submitted models are already good and not much room is left for refinement [30]. To assess the impact of refinement on surface binding properties we considered these refinement targets separately. Using the sequences provided by the CASP12 website for the refinement targets, we identified 31 structures by BLAST searches of the refined sequences against the PDB; the query coverage and percent identity recorded correspond to the structure listed as the PDB ID in Table 2SI. We note that the regular and refinement targets are identified as T0 and TR, respectively, in front of the target ID. A total of 11 CASP12 refinement targets were removed from the analysis for having no experimental structure deposited in the PDB (TR874, TR875, TR876, TR881, TR890, TR896, TR898, TR901, TR905, TR913, TR947). To focus on the well-defined regions of the target structures, CASP organizers further trimmed many of the refinement targets. For example, the regular target T0862 has 239 residues (see Table 1SI), but the refinement target TR862 has only 101 residues (Table 2SI). Thus, the experimental structure (Chain B of the PDB structure 5J5V) was trimmed accordingly for this case, and all similar cases.

### 2.3 Selection of homologs for the analysis of surface binding properties

To establish a baseline for how surface properties are conversed between homologs, we searched for homologs of the CASP12 targets published in the PDB. We performed this search by running mmseqs2 on the CASP12 reference PDB (listed in Table 1SI), and selected structures with high sequence similarity (generally >90%), and good query coverage [31]. We manually checked and trimmed the structures to make sure they properly aligned to the target X-Ray PDB structure. In these manual alterations, homologous structures for targets T0866, T0892 and T0914 were trimmed to match the target sequence. One homologous structure for T0859 (5LQP) was removed due to low resolution – it is a CryoEM reconstruction of a homo-180-mer with 6 Å resolution. Additionally, one homologous structure for target T0907 was removed (6EY6_A) because the structure was missing one domain of the protein (residues 217–319). Homologous structures for T0948 were also removed (5TIB_A, 5TJ2_A, 5TJ4_A) because they were missing coordinates for residues 1372–1382, a loop critical for binding site detection. Finally, for T0866, a homolog (6TS8_A) was removed because it has a cysteine mutation (177G to 177C) that significantly impacted binding site detection near the mutation [32]. In total, we evaluated 51 homologs for CASP12 targets in the PDB.

### 2.4 Targets for testing protein–protein interactions

To investigate whether the models can be used to reproduce observed protein–protein interactions we selected the 17 targets that interacted with a different chain - wherever possible, we used the PDB structures shown in Table 1SI. These targets included 6 homo-dimers, 3 homo-trimers, 2 homo-tetramers, 4 hetero-dimers, one hetero-trimer, and one hetero-tetramer with the A2B2 stoichiometry. In each case we focused on the interaction between the chain that defined the target and the rest of the interacting protein in the PDB structure, resulting in 23 binary interactions (see Table 3SI). The same method was applied to the refinement targets, which provided binary interfaces for an additional 14 targets.

### 2.5 Generating binding fingerprints using the FTMap program

The FTMap program globally samples the conformations of 16 different molecular probes (ethanol, isopropanol, isobutanol, acetone, acetaldehyde, dimethyl ether, cyclohexane, ethane, acetonitrile, urea, methylamine, phenol, benzaldehyde, benzene, acetamide, N,N-dimethylformamide) on a dense grid around the protein, generating over 70,000 conformations for each probe type [26], [33]. FTMap has been developed as a computational analog of the experimental technique called Multiple Solvent Crystal Structures (MSCS), which involves determining X-ray structures of a target protein in aqueous solutions containing high concentrations of organic co-solvents, and then superimposing the structures to find consensus binding sites that accommodate a variety of the organic probes. Ringe and coworkers have shown that such consensus sites identify hot spots that provide major contributions to the binding free energy, and that the number of probes interacting with specific regions of the proteins represent the importance of the region for ligand binding [16], [17]. The fast Fourier transform (FFT) correlation method makes the global grid sampling computationally feasible [33]. The energies of the protein-probe interactions are evaluated using an energy function that includes molecular mechanics, continuum electrostatics, and structure based empirical energy terms. For each probe type the 2000 lowest energy docked structures are retained for further analysis. The retained structures are minimized using the CHARMM energy function [34] with the analytical continuum electrostatics (ACE) term [35], allowing for the flexibility of the probes and of protein side chains. The resulting probe positions are used both for describing the binding properties of the entire protein surface and for determining binding sites (see below). For each structure, the binding fingerprint is calculated as the number of probe-residue contacts associated with each residue, where a residue and a probe are considered to be in contact if any probe atom is within 3 Å of any atom of the residue. Since the experimental structure deposited in the PDB often varies slightly (typically by a few residues) from the given CASP sequence, we align the two sequences using the Needleman-Wunsch algorithm [36] with the Gonnet substitution matrix [37]. Gaps are represented in the fingerprint with zero probe-residue contacts. Two fingerprint vectors are compared simply via calculation of the Pearson correlation coefficient (denoted as PCC in the rest of the paper).

### 2.6 Determining binding hot spots and ligand binding sites

After calculating the binding fingerprints, the 2000 lowest energy docked positions generated by FTMap for each of the 16 probes are clustered, and the clusters are ranked on the basis of the average energy [26]. The six lowest energy probe clusters of each probe type are retained, and clustering is performed once more on the clusters of all probe types to form consensus clusters, which are ranked by the number of included probe clusters. It was shown that such consensus clusters identify the locations of binding hot spots, which are the regions with the highest contribution to the binding free energy [26], and provide information on the potential druggability of the protein [38]. Although this algorithm includes heuristic elements, its results have been rigorously tested in a large number of applications [39], [40], [41], [42], [43], [44], [45]. We note that the probes used by FTMap vary in size, and mostly consist of polar and nonpolar moieties [26]. It was observed that the ligand binding regions generally exhibit mosaic-like arrangement of polar and nonpolar patches that enable the binding of multiple probes [41], and hence the clustering of probe clusters mostly occur at such locations, substantially reducing the possibility of false positives [26]. We have also shown that the 16 probes we currently use provide reliable information, and additional probes did not further improve the prediction of binding hot spots.

Merging hot spots that are close to each other yields high accuracy prediction of ligand binding sites. This algorithm was implemented in the FTSite server and is adapted for this study [46]. In this analysis, the binding sites are simply ranked by the number of probe clusters in the binding site. Nine CASP12 targets in the PDB have been either co-crystallized with small ligand molecules or information on binding site residues was available, and we will use the mapping to determine whether the binding sites of these ligands can be found both in the X-ray structures and in the models. We note that the results presented in this paper were obtained by a command line version of the FTMap and FTSite programs [47], and the specific clustering and ranking of the binding sites may slightly differ from those produced by the FTMap and FTSite web servers. The command line version was used to accommodate the large number of mapping jobs required to assess the CASP12 targets, their homologs, and their 5 highest ranked models, 543 structures in total.

### 2.7 Docking of small ligands to proteins

As will be discussed, structures co-crystallized with small ligands were available only for a few targets. Nevertheless, we explored docking of such ligands to the X-ray structures and to the models of the receptor proteins to investigate the relationship between model quality and the accuracy of binding pose prediction. All docking calculations were performed by using AutoDock Vina 1.12 [15]. For each target we docked the ligand to the X-ray structure of the receptor extracted from the protein–ligand complex in the PDB, and to the 30 models with the highest GDT_TS scores. In preparation for the docking, all inorganic molecules, such as water molecules or ligands, were removed, and the protein structures were prepared using AutoDockTools (ADT) 1.5.7 [48] to add hydrogen atoms and to create PDBQT format files required for AutoDock Vina. Ligand structures for the docking simulations were generated based on the SMILES description given in the PDB, and the ligands were considered flexible - hydrogen atoms were added by using OpenBabel 2.4.0 and then manually adjusted to pH 7 [49]. The resulting structures were processed by ADT to generate PDBQT format files that were used in all docking calculation for the given target. Docking was restricted to a box with 15 Å sides, centered at the geometric center of ligand atoms in the crystal structure. However, it is generally also possible to define the “docking box” around the center of the strongest hot spot. Indeed, as will be further discussed, for all targets selected for ligand docking in this paper the ligand binding site was identified as the top binding site by FTMap. Thus, for cases where the ligand-binding location is unknown it would be useful to center the box over the predicted FTMap binding sites. AutoDock Vina parameters were set to default, apart from the exhaustiveness parameter that was increased from the default value of 8–10 to search the space in the box more exhaustively. The program samples and clusters the binding poses of the ligand, and returns the representative structure for each cluster. We used DockRMSD [50] to calculate the RMSD values of the representative structures by taking the ligand in the X-ray structure for reference.

In addition to AutoDock Vina, the small ligands were also docked to the three targets using a template-based method called ClusPro LigTBM [51], which was one of the best performing methods in the blind small-molecule docking competition Drug Design Data Resource (D3R) Grand Challenge. The method is based on the observation that high resolution structures of all major protein families are available in the Protein Data Bank, and the active centers of many of these structures contain low-molecular-weight substrates, inhibitors, or other assorted ligands. This structural information is used to perform template-based pose prediction by searching the PDB for known complexes containing a ligand sharing common substructure with the target and bound to a remotely-homologous protein. The target ligand is initially positioned based on the template and then refined using Monte Carlo minimization with a molecular-mechanics force field.

### 2.8 Protein-protein docking by ClusPro

As described, we selected the complexes in the PDB that included any CASP12 target, and performed two types of docking calculations. First, we removed the chain representing the target, trimmed it to agree with the sequence of the target in CASP, and docked back to the rest of the complex. We used the biological assembly listed on the PDB website for the complex after confirming it with PISA (see Table 1SI). Each X-ray structure was docked twice. Once with the target chain as the ligand and the rest of the complex as the receptor, and then again with the target chain as the receptor and the rest of the complex as the ligand. The better of the two results was retained. Second, the better performing combination was used to carry out the same calculations using the top 5 models of the target rather than the X-ray structure of the target chain.

The docking was performed using our server ClusPro [13], which includes the program PIPER as its docking engine [52]. PIPER performs rigid body docking in the 6D space of rotations and translations. The 70,000 rotations we consider correspond to about 5 degrees in terms of the Euler angles. The step size of the translational grid is 1 Å. Although the program evaluates the energy for billions of conformations, this can be efficiently done using fast Fourier transforms. For this paper we used the balanced energy coefficients as implemented in PIPER [52]. The 1000 lowest energy docked structures were clustered using pairwise interface root mean square deviation (IRMSD) as the distance measure. The structures at the centers of the 10 largest clusters are considered as the predictions of the complex, and are refined by local energy minimization using the CHARMM potential [34].

The quality of docking results was assessed using the measures introduced for CAPRI (Critical Assessment of PRedicted Interactions), the community-wide docking experiment similar to CASP [27]. In CAPRI three related parameters were used for assessing a model: the fraction of native contacts, the backbone root mean square deviation of the ligand (LRMSD) from the reference ligand structure after superimposing the receptor structures, and the backbone RMSD of the interface residues (IRMSD) [28]. Based on these measures, the organizers of CAPRI defined four categories of accuracy, which are incorrect, acceptable, medium, and high accuracy. More recently a continuous score called DockQ was developed that encapsulated the above three measures [29]. The DockQ values range from 0 to 1, where a value exceeding 0.80 implies high accuracy, between 0.80 and 0.49 medium accuracy, and between 0.49 and 0.23 acceptable accuracy. DockQ has been widely accepted and hence is used in this paper.

## 3 Results

### 3.1 Analysis of binding surface conservation using binding fingerprints

**Fig. 1:**
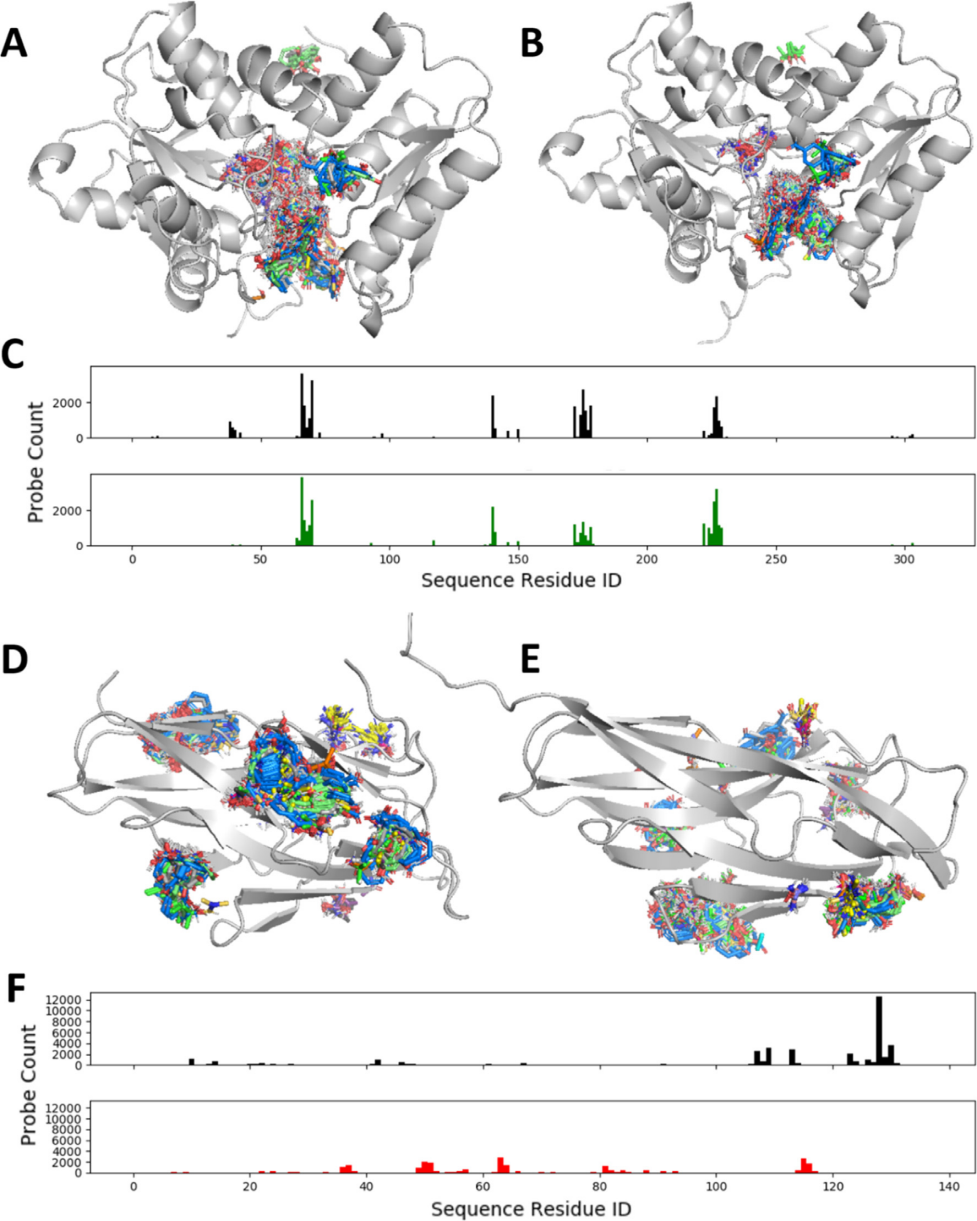
Examples of mapping CASP12 targets. (A) Mapping of the X-ray structure PDB ID 5J5V, chain A, of target T0861. The protein is shown as gray cartoon, and clusters of probes molecules as colored sticks. (B) Mapping of the best model T0861TS359_5 of target T0861 with GDT_TS = 99.04. Notation is same as in (A). (C) Binding fingerprints for the X-ray structure PDB 5J5V_A (black) and the model T0861TS359_5 (green). The binding fingerprint Pearson correlation coefficient (PCC) between the two fingerprints is 0.87. (D) Mapping of the X-ray structure PDB 5AOZ, chain A, of target T0921. (E) Mapping of the best model T0921TS220_2 of target T0921with GDT_TS = 70.65. (F) Binding fingerprints for the X-ray structure PDB 5AOZ_A (black) and the model T0921TS220_2 (red), with PCC = −0.08. (For interpretation of the references to color in this figure legend, the reader is referred to the web version of this article.)

Binding fingerprints were generated using FTMap for the CASP12 targets with experimentally determined structures deposited in the protein data bank (Tables 1SI and 2SI), and for the top 5 ranked models for each target. The similarity between the target structure and each model was assessed by calculating the Pearson correlation coefficient (PCC) of the two vectors. An example of a target with highly correlated predicted structures is T0861. Fig. 1A and B show, respectively, mapping results for the target protein, chain A of cysteine synthase A of *escherichia coli* 536 (PDB ID 5J5V_A) [53], and that of the model T0861TS359_5, which is ranked second by CASP but has the highest binding fingerprint correlation with the X-ray structure (PCC = 0.87). The binding fingerprints for the target and the model in Fig. 1C show the high level of binding surface similarity. As shown in Fig. 1A, in the X-ray structure most of the probes fall into one large binding area on the protein. Part of this region is the active site of the enzyme, which binds the C-terminal Gly-Tyr-Gly-Ile peptide tail of another protein, tRNA nuclease CdiA (T0862, PDB ID 5J5V_B) [53]. The other part of the binding region extends beyond the interface with T0862 and accommodates an L-peptide linker, a covalently bound ligand [53]. The top 5 models for T0861 have very similar binding fingerprints to that of the X-ray structure, with the average PCC of 0.79, and thus this serves as an example where the surface properties of the predicted structures closely match the real structure. In contrast, the mappings of the target T0921 and its model (Fig. 1D and E, respectively), show a case where all five top ranked models have very poor binding fingerprint correlation with the experimental structure (Chain A of 5AOZ), with the average PCC being 0.09. The sequence for T0921 encodes a cellulosomal scaffold protein that presents as a monomer, and the protein folds as an immunoglobulin-like β – sandwich domain. The mapping places several moderately strong hot spots in a shallow cavity between two β strands in the X-ray structure. In the models a slightly mispredicted loop expands into this cavity and excludes the probes, which become distributed in many places all over the protein surface, resulting in the very poor correlation shown in Fig. 1F. The highest ranked model by CASP (T0921TS220_2) for T0921 (GDT_TS = 70.65) has the lowest mapping correlation, PCC = −0.08, and the binding fingerprint of the model is clearly very different from that of the X-ray structure.

**Fig. 2:**
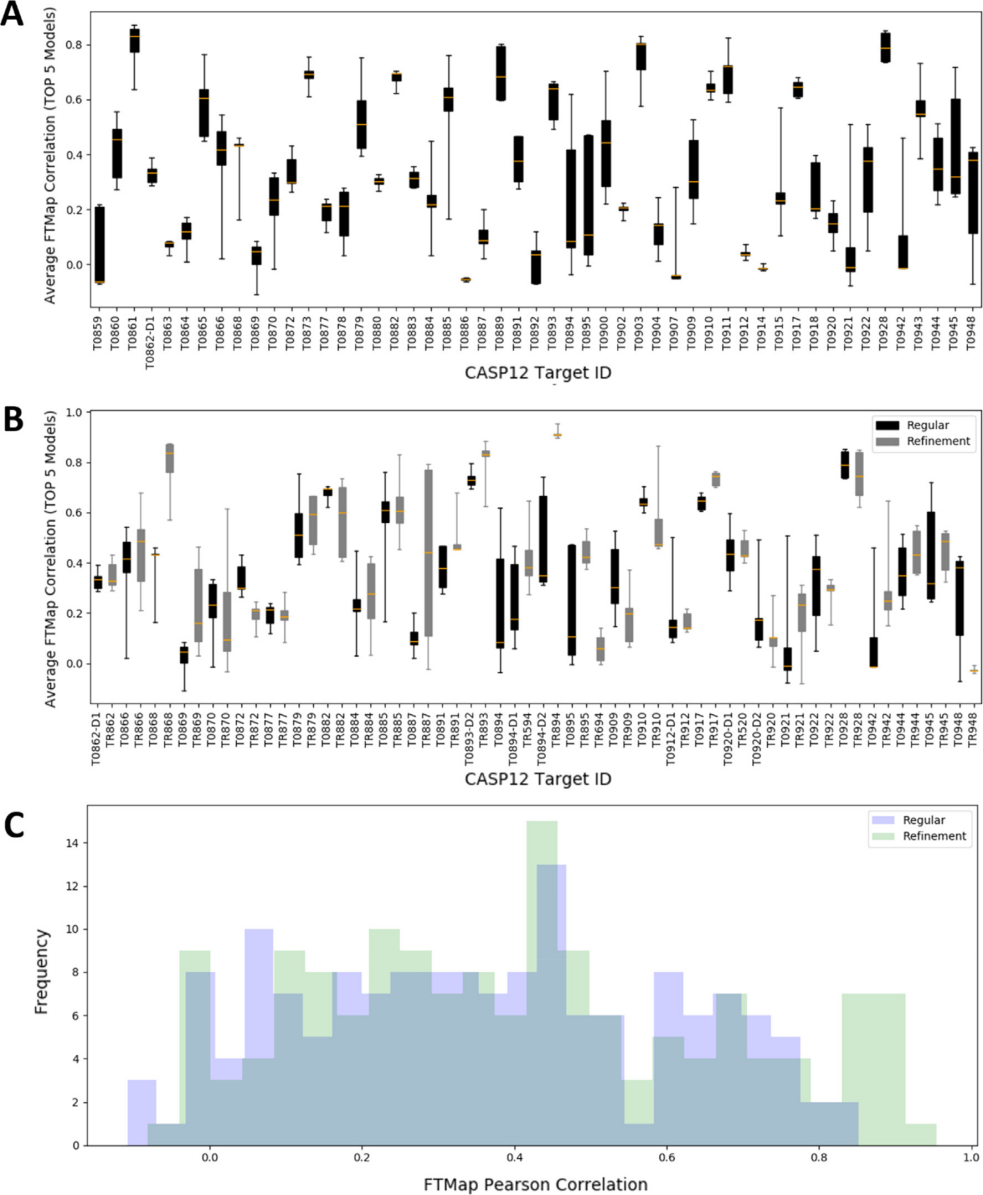
FTMap binding surface property conservation for CASP12 targets. (A) Box plots of binding fingerprint Pearson correlation coefficient (PCC) values for the top five models of the 51 CASP12 regular targets. The yellow center-hash indicates the average PCC and the whiskers extend to capture the range of the data. (B) Box plots of binding fingerprint PCC values for the top five models of the 31 CASP12 targets that have both regular and refinement versions. The PCC values for regular and refinement targets are shown as black and gray boxes, respectively. (C) Histograms of PCC values for regular (light blue) and refinement (light green) targets. (For interpretation of the references to color in this figure legend, the reader is referred to the web version of this article.)

Fig. 2A shows the box plot of the binding fingerprint PCC values for the top five models of the 51 CASP12 regular targets, ranging from −0.11 to 0.87, with an overall mean PCC of 0.33 and standard deviation of 0.24. The average GDT_TS and PCC values for the top five models of each target are listed in Table 4SI. An interesting question is whether refinement can improve the prediction of binding properties and thus improve PCC values. As we described, the refinement targets in CASP were based on regular targets, with the initial models released for refinement. Fig. 2B shows the average PCC values of the top 5 models for the 31 refinement targets and for the corresponding regular targets side-by-side. The average GDT_TS and PCC values for the top five models of the refinement targets are listed in Table 5SI. These results show a minor overall improvement in the PCC values for the refinement targets (mean PCC = 0.40, std = 0.27) relative to their regular counterparts (mean PCC = 0.36, std = 0.24). According to a *t*-test, the difference is not significant at the 5% level (p = 0.104), and the PCC histogram is only slightly shifted toward higher values (Fig. 2C). Thus, while there was only mild improvement collectively, some targets improved significantly with refinement (i.e. 868, 887, 891, 893, 894, 942), and others got worse (i.e., 872, 910, 948).

**Fig. 3:**
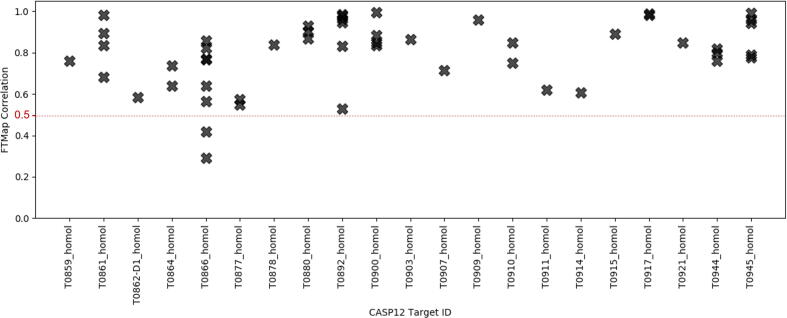
Binding fingerprint PCC for CASP12 targets with multiple X-ray structures in the Protein Data Bank.

Before further discussion of similarity between X-ray structures and models in terms of their binding surfaces it is important to establish a baseline for how well the fingerprints for independently determined experimental structures of the same protein correlate. Therefore, we calculated the pairwise binding fingerprint correlations for all CASP12 targets that had two or more experimentally determined structures deposited in the PDB (see Fig. 3 and Table 6SI). Between such structures the average binding fingerprint PCC is 0.80 with standard deviation of 0.16 across the 51 homologs evaluated. In the interest of creating a conservative guideline for homolog surface property correlation, we suggest that binding fingerprint PCC ≥ 0.5 represents general surface property conservation and is observed across most homologs in the PDB, as 49 of the 51 homologs we evaluated meet this threshold. Notably, the two homologs for target T0866 that fall below this threshold (6ZY2_A with PCC = 0.29 and 6ZY9_A with PCC = 0.42) appear very similar to the T0866 reference PDB (5UW2), both with GDT_TS > 85, but show some different side chain orientations that appear to have a large impact of the surface properties (see Fig. 1SI). We note that such changes are biologically relevant, and we have observed in the past that changes in protein conformation can dramatically affect the surface binding properties (i.e. a kinase DFG-in versus DFG-out conformation). However, across most of the CASP12 homologous structures, we see good conservation of binding sites, and therefore propose to roughly equate this observation with a criterion of binding fingerprint PCC ≥ 0.5. Below this threshold, we observe significant differences in the binding properties of the structures, and while such differences may be explained by biological conformational changes, they do not seem common among the CASP12 targets evaluated here. While the average PCC = 0.8 among homologs is higher than correlations between X-ray structures and models, for 24 of the 51 regular targets and for 19 of 31 refinement targets the best PCC values among the top 5 models exceed 0.5 (see Table 4SI and Table 5SI), demonstrating fairly high level conservation of the binding surface in these models.

**Fig. 4:**
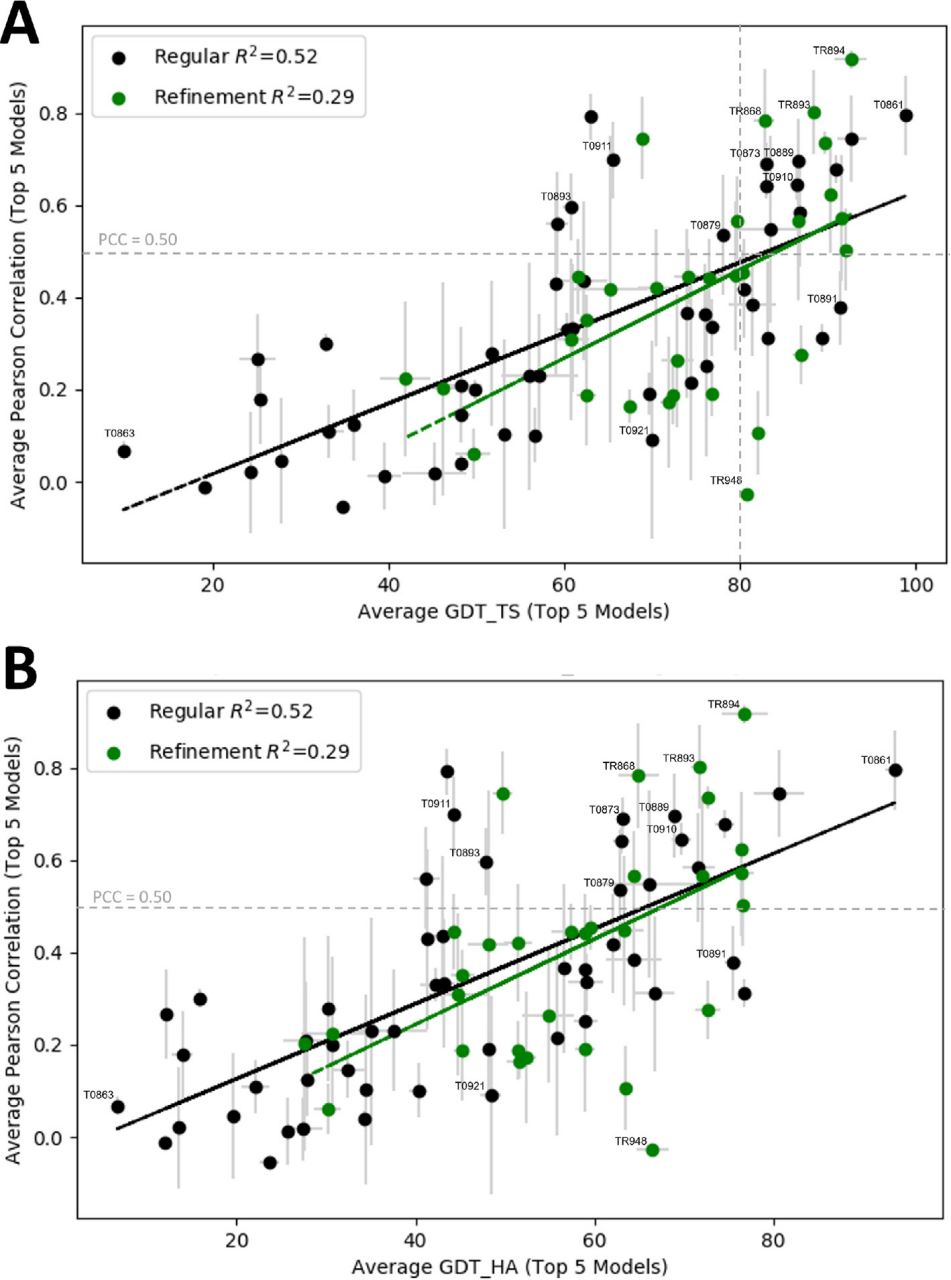
Binding fingerprint PCC values versus GDT Scores. (A) Average PCC values for the 5 top ranked regular and refinement models vs. GDT_TS. (B) Average PCC values for the 5 top ranked regular and refinement models vs. GDT_HA. Regular targets average PCCs are shown as black dots, standard deviations are shown as gray lines, and the linear regression is shown as a black line. Refinement targets average PCCs are shown as green dots, standard deviations are shown as gray lines, and the linear regression is shown as a green line. (For interpretation of the references to color in this figure legend, the reader is referred to the web version of this article.)

One of the main goals of our study is to determine how the accuracy measures used by CASP relate to surface binding properties described in terms of PCCs. Fig. 4A and B, respectively, show the PCC values for both the regular and the refinement CASP12 targets as functions of GDT_TS and GDT_HA, averaged over the top five models for each target. For the regular targets the PCC values are correlated with both GDT_TS and GDT_HA (R^2^ = 0.52 in both cases). Since the refinement targets are in a narrower range of the quality measures, the PCC values have somewhat weaker correlations, R^2^ = 0.29 for both GDT_TS and GDT_HA. The two plots are very similar, and hence we focus on the dependence of PCC values on GDT_TS as shown in Fig. 4A. The most important observation is that to reach PCC = 0.5, the low threshold observed for different structures of the same proteins, it is necessary to generate models with GDT_TS = 80 or higher. About 50% of models above this threshold have PCC > 0.5. Among the targets with the highest PCC values are the already discussed T0861, which has a deep active site pocket that also binds a covalent ligand, and is reproduced very well in all models. As will be discussed, targets T0893/TR893 and T0873 also have bound ligands [8], and although ligand information is generally not used in the modeling of the proteins, the surface properties are easier to model for proteins that have well-defined ligand binding sites. In TR868 and TR894 the most likely explanation for the high PCC values is that these proteins have large concave binding sites primarily determined by regular secondary structures that are well reproduced in the models. In TR868 (chain A of PDB ID 5J4A) the target is a tRNA nuclease CdiA [54]. The main hot spot is in a deep cavity, surrounded by four α-helices and a long loop. The pocket provides the binding site for the protein CdiI [54], forming a complex with the target in 5J4A. In the top 5 models the four helices essentially overlap with the ones in the X-ray structure, resulting in both high GDT_TS and PCC values. Although the loop partially covers the site in the models, it does not prevent the binding of the small probes. In TR894 the target is a very simple small domain, which consists of a single α-helix on top of a 3-strand β-sheet, and the only strong binding hot spot is located between these secondary structure elements. The site is correctly identified in all good models, which again leads to both high GDT_TS and PCC. However, GDT_TS > 80 does not necessarily imply that the models correctly capture the surface binding properties of the X-ray structure, and Fig. 4A shows several targets that have only models with PCC values much lower than 0.55 in spite of the high GDT_TS. As we just discussed, targets with well-defined ligand binding sites or sites defined by invariant secondary structure elements are likely to have models with PCC > 0.55. In contrast, targets that have binding sites defined by flexible loops or a largely featureless surface are likely to have models with low PCC values. For example, in TR948 the probes bind in a deep pocket at the end of three helices among loops. In the models, the helices are slightly extended, and some of the incorrectly predicted loops cover the entrance of the pocket, which becomes inaccessible. Although the average GDT_TS = 80.8 is very high, almost no probes can bind at the main binding pocket and hence distribute in various shallow pockets on the protein surface, resulting in the average PCC of −0.03.

**Fig. 5:**
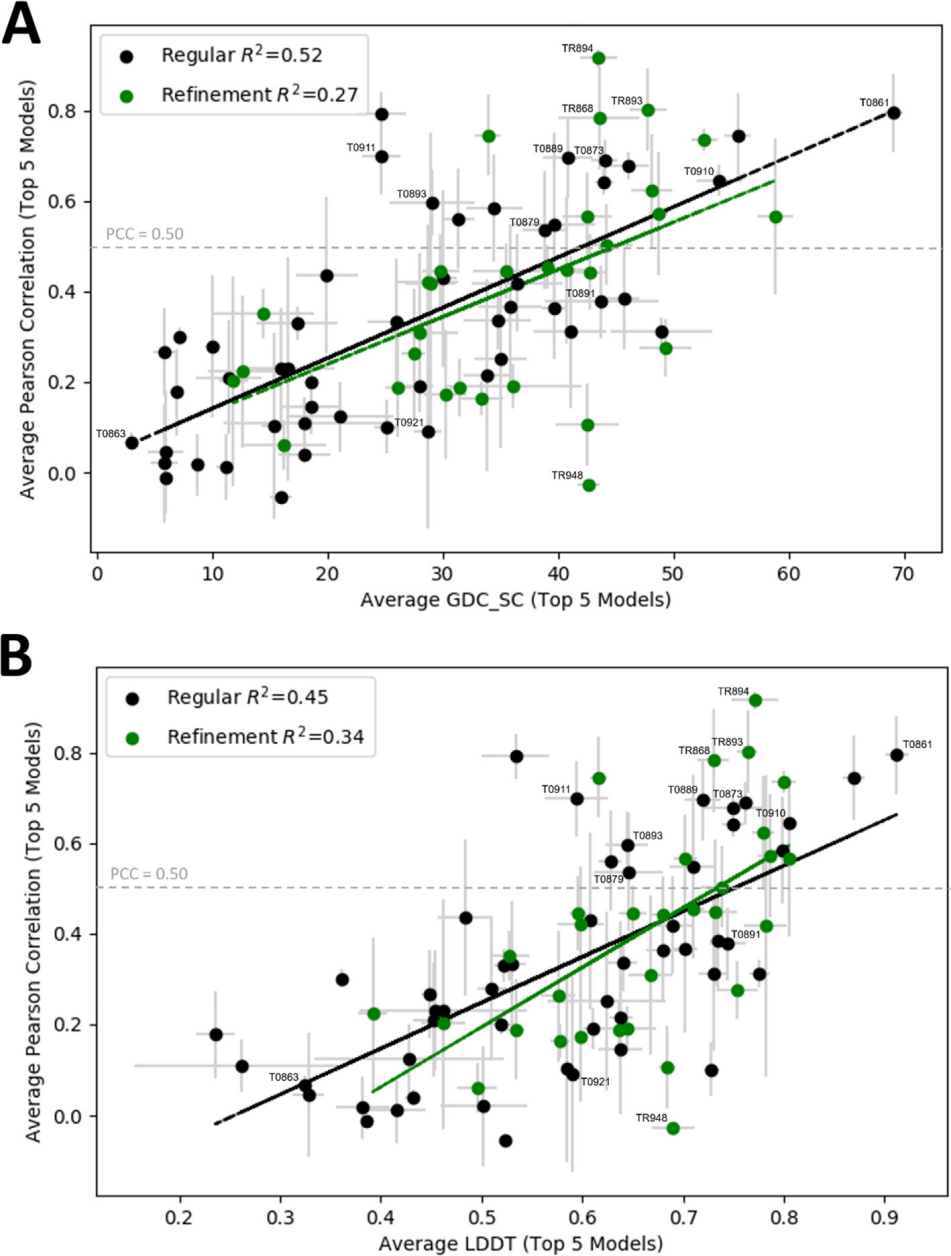
Binding fingerprint PCC values versus GDC_SC and LDDT values. (A) Average PCC values for the 5 top ranked regular and refinement models vs. GDT_SC. (B) Average PCC values for the 5 top ranked regular and refinement models vs. LDDT. Regular targets average PCCs are shown as black dots, standard deviations are shown as gray lines, and the linear regression is shown as a black line. Refinement targets average PCCs are shown as green dots, standard deviations are shown as gray lines, and the linear regression is shown as a green line. (For interpretation of the references to color in this figure legend, the reader is referred to the web version of this article.)

While GDT_HA is a more demanding quality measure than GDT_TS, Fig. 4A and B show that the PCC values depend very similarly on the two measures, and hence we focus on GDT_TS, which is used for ranking the CASP models. We have also studied the dependence of PCC values on many different quality criteria introduced for CASP, and expected that some of the measures will provide additional information. One such measure is GDC_SC (Global Distance Calculation for sidechains), a GDT-like metric, which uses a characteristic atom near the end of each side chain type instead of α-carbons used in GDT_TS and GDT_HA, and is defined by GDC_SC = 2*(k*GDC_P1 + (k − 1)*GDC_P2 … + 1*GDC_Pk)/(k + 1)*k, k = 10, where GDC_Pk denotes the percent of residues under the distance cutoff ≤ 0.5 kÅ. This measure is potentially relevant since the surface binding properties depend on side chain conformations. Nevertheless, the PCC versus GDC_SC plot (see Fig. 5A) is similar to the PCC versus GDT_TS plot, indicating that the primary determinant of model quality is the accuracy of the backbone, even when considering the prediction of surface properties. Another local measure is the LDDT (Local Distance Difference Test) score [55]. The LDDT values were computed using the following procedure: a list of pairwise nonbonded distances was generated from the target protein structure. For each atom i, all atoms j not part of the same residue as i and lying within 5 Å from i were considered as interactions partners of i. The cumulative list of i-j interactions stemming from all atoms in the experimental protein structure was taken as reference against which to score predictions. Specifically, interaction distances in the protein structure were compared with distances between corresponding atoms in the predictions. If the difference between the two distances was below a defined threshold, the interaction was considered to be preserved in the prediction. The final LDDT-all score was computed by averaging the fraction of correctly modeled interactions for the following four distance difference thresholds: 0.5, 1, 2, and 4 Å (the same thresholds as GDT_HA). Although the concept of LDDT substantially differs from that of GDT_TS and GDT_HA, according to Fig. 5B the relationship between the binding fingerprint PCC and LDDT provides limited additional information. Tables 7SI and 8SI, respectively, list the GDC_SC, LDDT, and binding fingerprint PCC values for the top 5 ranked models of the CASP12 regular and refinement targets.

### 3.2 Conservation of binding sites in predicted structures

**Fig. 6:**
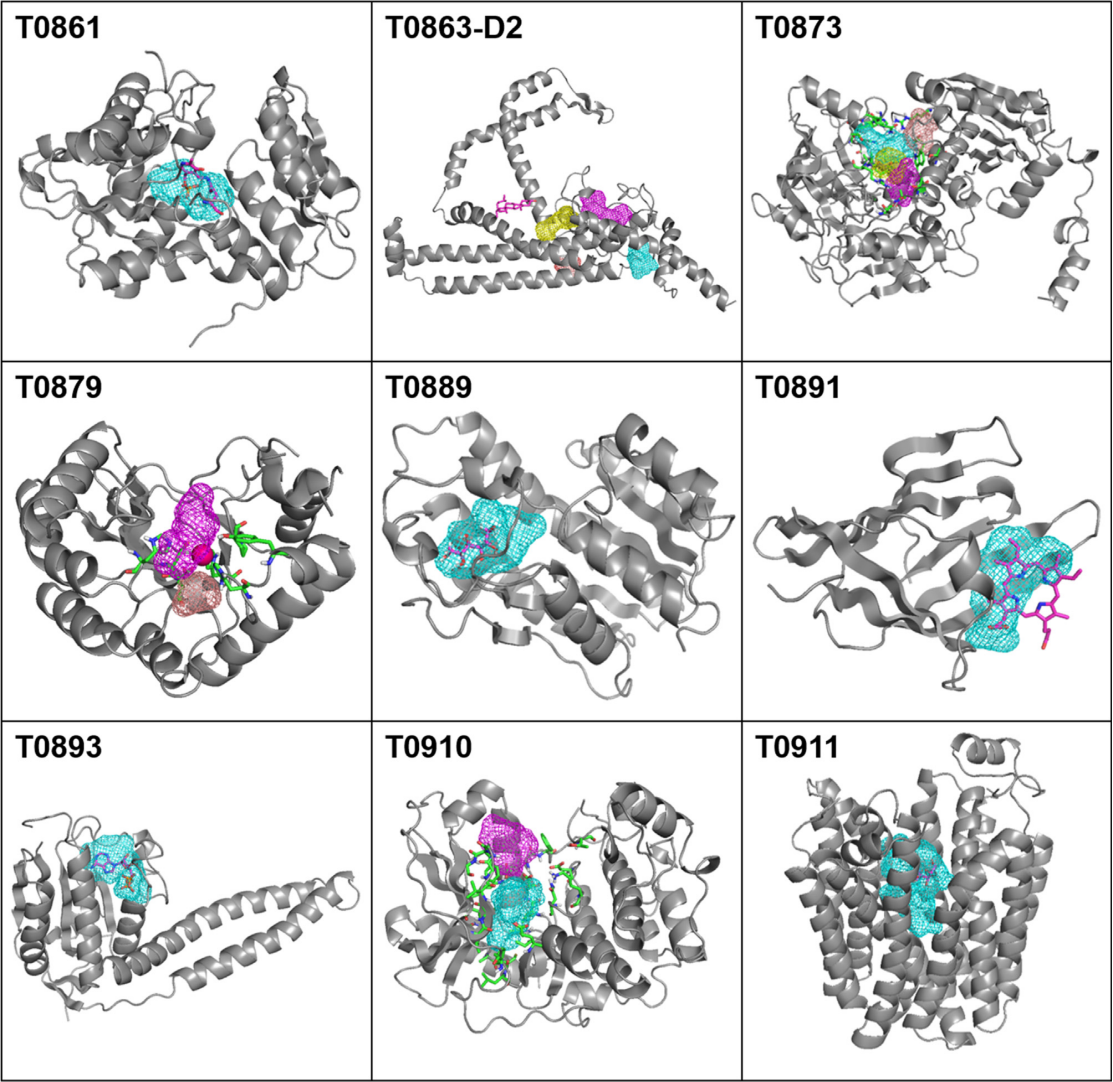
Binding sites of CASP12 targets with ligands. The X-ray structure of each target (PDB IDs and ligand IDs are listed in Table 1) is shown as a gray cartoon, and the bound ligand is shown in pink sticks. In target T0879 the pink dot represents the bound Zn^2+^ ion. The relevant ligand-binding sites are shown as mesh and color-coded according to their rank (cyan > magenta > yellow > salmon). For targets with no ligand shown and T0879, the *a priori* known binding site residues are shown as green sticks. (For interpretation of the references to color in this figure legend, the reader is referred to the web version of this article.)

Previous functional assessment of CASP12 targets by Altman and colleagues [8] identified nine targets where a ligand was co-crystalized with the protein, and therefore had a known ligand-binding site. For two targets (T0873 and T0910) the ligand was not co-crystalized with the protein in the PDB structure currently available, and the binding site was determined based on the binding residues identified by the authors of the structures and described by Liu et al. [8]. FTMap was applied to all structures after removing the ligands. The mapping, followed by the clustering of the probes and probe clusters (see Methods) correctly identified the ligand binding site as the strongest or second strongest hot spot region in eight of the nine targets (see Table 1, Fig. 6, and Table 9SI). The exception, target T0863 (PDB ID 5SY1_A) encodes a transmembrane protein, STRA6 [56], and provides a challenging case both for structure and binding site prediction, due to the hydrophobic nature of the protein and the binding pocket. All models are very poor with the best GDT_TS = 10.22, and average GDT_TS = 9.86 (Table 3SI). With such poor models we cannot expect meaningful binding site prediction. In addition, since FTMap was developed for the analysis of soluble proteins [26], it does not identify the cholesterol binding site located in the protein-membrane interface [56]. However, in the other 8 targets FTMap placed a substantial number of probe clusters at the ligand binding site (note that in Table 1 “Probes” actually accounts for the number of probe clusters). Since FTMap uses 16 different probe types and retains the six lowest energy clusters for each [26], the maximum number of probe clusters is 96, thus in most targets the ligand binding site attracted close to 50% of all probe clusters (see Table 1). In seven targets the site was the strongest (had the highest number of probe clusters among all sites) and the second strongest in T0879 co-crystallized only with a Zn^2+^ ion. In Table 1 we show the conservation of ligand binding sites in the models in terms of an overlap measure, calculated as the total number of probe clusters in the site found by mapping the model, divided by the total number of probe clusters in the X-ray structure found in the binding site overlapping with the ligand. Although the overlaps vary among the top five models, the level of conservation is fairly high. Fig. 6 shows each of the nine targets as a gray cartoon and the predicted ligand-binding site as mesh, with the rank of the site indicated by the color scheme shown in the figure. As mentioned, for target T0863 the predicted binding sites do not overlap with the bound ligand, and for targets T0873 and T0910 no ligand-bound structure is available in the PDB, but information was available on the ligand binding residues, shown as green sticks in Fig. 6 [8]. Table 1 also shows that well-defined ligand binding site can be identified even at moderate GDT_TS values around 60, since the probes tend to cluster in large cavities. In Table 9SI we list the residues within 5 Å of the probes that define the binding site.

**Table 1:** FTMap analysis of structures with known ligand binding sites.

target	PDB ID	ligand	average GDT_TS	average PCC	X-ray structure		probe cluster overlap (%) with binding site				
					rank	probes^a^	top 1	top 2	top 3	top 4	top 5
T0861	5J43_A	LLP^b^	98.77	0.85	1	44	38.02	46.39	31.56	37.26	38.02
T0863	5SY1_A	CLR^c^	9.86	0.07	–	0	0	0	0	0	0
T0873	6DA6_A	FMN^d^	82.93	0.69	1	36	56.19	54.12	51.55	51.55	57.22
T0879	5JMU_A	ZN	78.09	0.54	2	22	89.91	73.39	80.73	51.38	82.57
T0889	5JO9_A	SOR	86.69	0.70	1	43	55.98	54.07	46.41	44.98	54.07
T0891	4YMP_A	HEM	91.34	0.38	1	41	0	69.96	60.54	83.41	0
T0893	5IDJ_A	ADP	60.74	0.60	1	51	84.42	32.25	52.9	31.16	47.1
T0910	6BDL_A	ANP^d^	86.49	0.65	1	38	49.21	63.49	74.6	55.03	68.78
T0911	6E9N_A	GCO	65.99	0.74	1	84	39.82	37.14	16.33	3.58	12.98

a The number of probe clusters in the binding site.

b L-peptide linking (covalent ligand).

c Binding at the protein-membrane interface.

d No ligand in the PDB structure, Binding site residues are based on information from the author of the target.

**Fig. 7:**
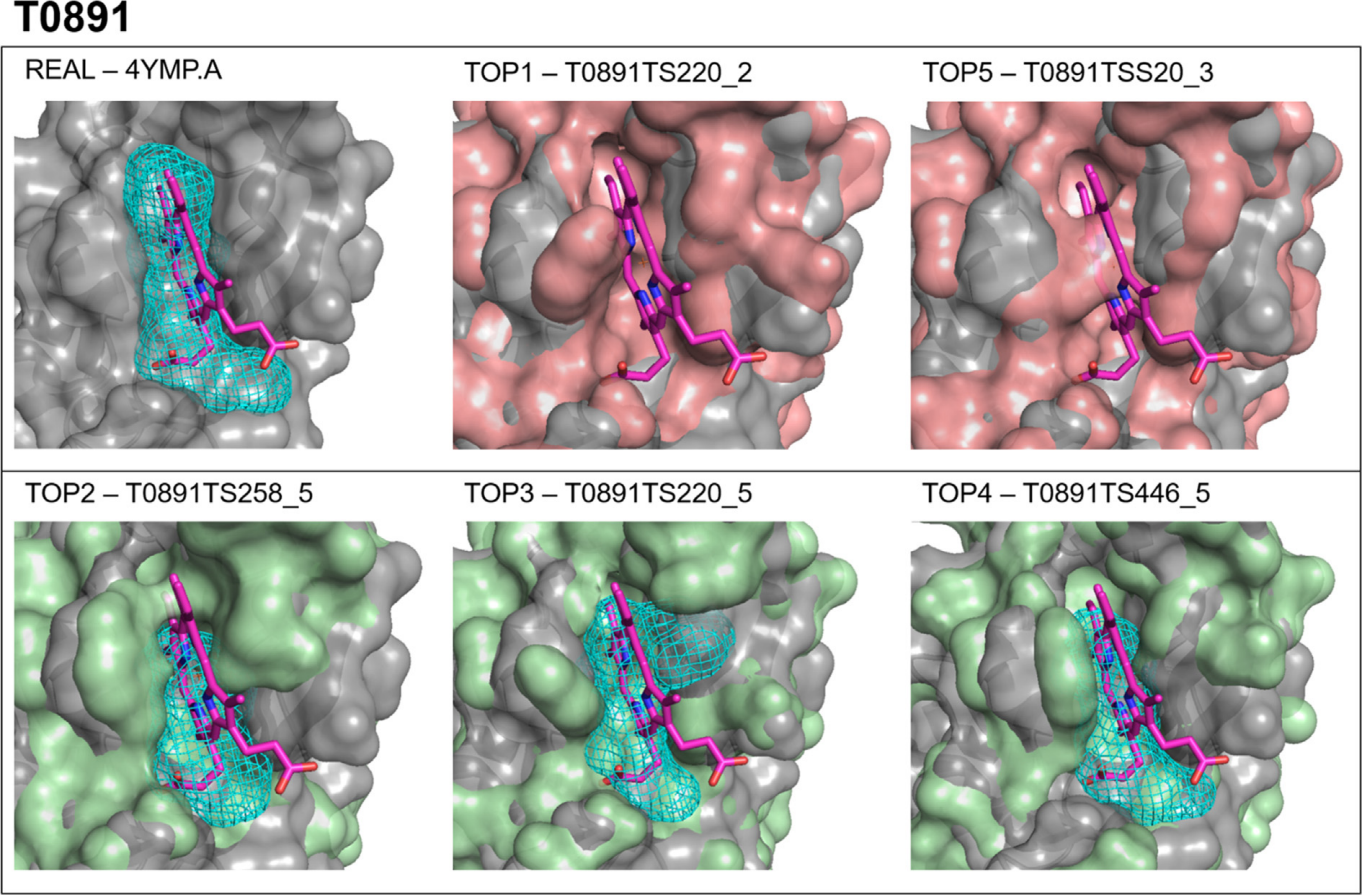
Protein mapping results of target T0891 structures, with heme overlaid. The X-Ray structure (PDB ID 4YMP) is shown in gray, and is co-crystalized with heme, shown as pink sticks. The structure of the model (red/green) is overlaid along with the binding site identified in the model, and the placement of the heme molecule, for reference. (For interpretation of the references to color in this figure legend, the reader is referred to the web version of this article.)

According to Fig. 4A, GDT_TS > 80 is required for PCC > 0.5, but a high GDT_TS does not guarantee a high PCC, which is lower than 0.5 for about half of such targets. An example is target T0891 (PDB ID 4YMP), a bacillus anthracis Hal NEAT domain in complex with heme. Although the average GDT_TS for the top five models is 91.34, the average PCC value is only 0.38 (see Table 1SI and Fig. 4A). The low PCC values are, in part, due to changes in the heme binding site (discussed below), but are also due to the variations in surface properties due to changes in the conformations of two loops in this protein (residues 1–9 and 63–69), both of which have missing coordinates in the CASP12-given X-ray structure, 4YMP_A. Table 1 shows that mapping of the X-ray structure reveals a very strong heme binding site with 41 probe clusters, and the site is also identified in models ranked 2, 3, and 4. However the top 1 and top 5 models show no heme binding site at all, despite having very high GDT_TS scores (91.74 and 91.07, respectively). The top 5 models for target T0891 are very similar, so the stark contrast in outcome of binding site identification is surprising. However, the surface representation in Fig. 7 shows that the heme binding site is too narrow in the top 1 model, seemingly closing the site, which is completely closed in the top 5 model. Thus, even very subtle local differences in the binding pocket structure can change the potential strength of the site. Importantly, because binding pockets can change conformation, a closed binding site may still bind ligands, but without alternative experimental structures or molecular dynamics simulations we cannot comment on the existence of the binding site. The structure in T0891 was also given as a refinement target in CASP 12, and the top 5 refinement models have a profile similar to the unrefined target with all structures GDT_TS > 90, but models 2 and 3 have very low binding site overlap, 1.35% and 4.04%, respectively, whereas the other three predictions have substantial overlap, 27.35%, 65.02%, and 52.02%. Thus, both for the regular and the refinement targets multiple models provide a conformational ensemble that can be used to assess the availability of a binding site.

Protein mapping of the remaining seven holo targets show ligand-binding sites identified across all 5 top predicted models, see Table 1. While some model binding sites are weaker than the X-ray binding site, we expected the latter to have better defined binding sites since they were co-crystalized with the ligand. Importantly, the presence of these binding sites shows promise for the use of these high quality predicted structures, in the absence of an experimental structure, to identify druggable binding sites [38], particularly when an entire ensemble of models are available. However, as shown for target T0891, due to local conformational variations a strong binding site may be seen in some but not necessarily in all models. The conformational variation can substantially affect the docking of small ligands, to be discussed in the next section.

### 3.3 Docking of small ligands to proteins

Ligand docking calculations are meaningful only to three of the nine “holo” structures in Table 1. In fact, targets T0873 and T0910 have no co-crystallized ligands in the PDB, T0863 binds cholesterol at the protein-membrane interface and all models are of low accuracy, targets T0861 and T0891 have covalent ligands, the just discussed T0891 is a hemoprotein, with the iron of the heme group coordinated by a Tyr side chain, and the ligand in T0879 is a Zn^2+^ ion. Since we exclude these targets, docking is limited to targets T0889, T0893 and T0911. It is clear that no general conclusions can be based on such few cases. However, since docking of small molecules was explored in previous CASP functional evaluations, we include these calculations here.

Target T0889 (PDB ID 5JO9) is a complex of sorbitol dehydrogenase from thermostable *Bradyrhizobium japonicum* and sorbitol [57]*.* Sorbitol is a small molecule, and in such cases 2 Å RMSD is considered a good docking result. As shown in Table 10SI, for the X-ray structure the best RMSD is 2.04 Å, thus it is very close to the native, and this structure is among the five lowest energy docked structures provided by AutoDock Vina. The RMSD of the lowest energy (Top 1) structure is 2.21 Å. Similar accuracy is achieved for a number of models, with 3.04 Å as the average RMSD of the best structures. Nevertheless, there is considerable variation in accuracy among different models, and the RMSD of the best structure can be as high as 5.27 Å (Table 10SI). The crystal structure indicates that the bound sorbitol is stabilized via hydrogen bond interactions with the side chain of Glu150 and Ser140, and the carbonyl groups in the backbone of Pro183 and Gly184. All 30 models for this target have high GDT_TS (average value is 85.09), and the backbone and even the Ser140 side chain show only very small deviation from the X-ray structure. Thus, the variation in the position of the ligand is entirely due to the variation in the position of the Glu150 side chain, and it follows that there is no observable correlation between either the GDT_TS or the PCC values and the accuracy of the docking. This observation will be discussed further in this section.

Target T0893 is a complex of a bifunctional histidine kinase and ADP (PDB ID 5IDJ) [58]. ADP is much bigger than sorbitol and has five rotatable bonds. In addition, the binding site of the protein in the X-ray structure includes a bound magnesium ion, which is removed prior to docking. Nevertheless, docking to the X-ray structure yields 1.89 Å RMSD as the top (lowest energy) docked structure (Table 11SI). The high accuracy is mostly due to the fact that the kinase has a well-defined binding site, and ADP is stabilized by nine hydrogen bonds. However, the top 30 models have only the average GDT_TS of 58.0, with substantial variations of two loops around the binding site. Accordingly, the accuracy of the docked structures also vary. The average RMSD of the best structures is 4.1 Å, and the average RMSD of the lowest energy structures is 5.41 Å, both about is 1 Å higher than for target T0889. The RMSD of some lowest energy structures reaches 7.96 Å. Comparing these results with those for T0889 emphasizes that substantially higher accuracy of models leads to better docking results. However, the latter are affected by local conformational changes, and hence relatively small differences in GDT_TS do not predict the differences in docking results.

Target T0911 (PDB ID 6E9N) is a complex of an *escherichia coli* D-galactonate transporter and D-gluconic acid [59]. The models have the average GDT_TS of 63.79. The added complexity of the target is that the protein has been crystallized in the presence of nonyl beta-D-glucopyranoside molecules, and one of these compounds binds close to the D-gluconic acid ligand. The results of docking the ligand to the X-ray structure are poor, 5.29 Å and 6.69 Å for the RMSD of the best and the lowest energy structures, respectively (Table 12SI). The possible explanations for this large error are that the bound D-gluconic acid in the X-ray structure is stabilized by a single hydrogen bond (to the side chain of Asn 393), and that the docking was performed without placing the nearby nonyl beta-D-glucopyranoside molecule. The results of docking the ligand to the models are not much worse, or even slightly better, with the average a global measure of the conservation of binding properties, the RMSD of a ligand docked to different models depends on the conformations of the side chains around the ligand binding site. RMSD values of 5.97 Å and 6.63 Å for the best and lowest energy structures, respectively. However, since even docking to the X-ray structure does not work well, this result is not very informative. Tables 10SI, 11SI, and 12SI also show, respectively, the binding fingerprint PCC for each of the top 30 models for targets T0889, T0893, and T0911, relative to the X-ray structure. No correlation is seen between the best RMSD and the binding fingerprint PCC for T0889 or T0893, and the correlation is very weak for T0911 (R^2^ = 0.2844). The lack of correlation between these two metrics is expected, because while the binding fingerprint PCC is

In addition to AutoDock Vina, the ligands to targets T0889, T0893, and T0911 were also docked using the template based method ClusPro LigTBM [51], described in Section 2.7, and the results are also shown in Tables 10SI, 11SI, and 12SI. Note that docking by LigTBM requires only the sequence of the target protein, since the method itself finds homologous proteins with bound ligand to be used as templates. Thus, in its basic application mode the LigTBM results are independent of the CASP models. Therefore, while using AutoDock Vina we list docking results for the top 30 models, for LigTBM the we show only a single result for each target. These results emphasize that the accuracy of a template based method may substantially differ from target to target, and most likely heavily depends on the structures available as templates. In particular, LigTBM performs slightly better than AutoDock Vina for target T0889, it performs much better for target T0893, and worse for target T0911. However, as already noted, in this last case both methods yield very poor results.

### 3.4 Analysis of protein–protein interactions

As described in the Methods, we first docked the X-ray structures of each selected CASP12 target to its interacting partner in the PDB, and then replaced the target structure with each of the 5 top models and performed the same docking calculations. Table 2 shows the results for the regular targets. In the second column we show the PDB code of the multichain complex that includes the target. The next two columns identify the ligand and receptor as used in the process of docking. The two positions are not equivalent, since the receptor is fixed at the origin of the coordinate system, whereas the ligand is moved on rotational and translational grids. Depending on the arrangement which produced better docked structures of the X-Rays, the target can be either the ligand or the receptor, and is indicated in boldface fonts in Table 2, Table 3. In each docking we retain and analyze ten models, obtained as the centers of the ten largest clusters of docked structures generated by ClusPro. The quality of models is determined by calculating the DockQ score, and selecting the model with the highest score. Table 2 shows the rank of the best docked structure among the 10 options, along with the DockQ score. As mentioned, in terms of the categories introduced by CAPRI, DockQ ≥ 0.80 implies high accuracy, 0.80 > DockQ ≥ 0.49 medium accuracy, and 0.49 > DockQ ≥ 0.23 acceptable accuracy, whereas DockQ < 0.23 means that the structure is incorrect. The right side of Table 2 shows the results for replacing the target with each of the 5 best CASP12 models, thus performing five docking calculations. We select the model that yields the docked structure with the highest DockQ score, and list the rank of the docked structure among the 10 options for the selected model, as well as the DockQ score. The same calculations were carried out for the CASP12 refinement targets, with the results shown in Table 3.

**Table 2:** Docking of CASP12 regular cases, best DockQ score of top 10 ClusPro models.

CASP ID	Native	Docking of X-ray structures					Docking of models to X-ray structures				
	PDB ID	protein 1 ligand	protein 2 receptor	ClusPro rank	DockQ	accuracy	protein 1 ligand	protein 2 receptor	ClusPro rank	DockQ	accuracy
T0859-D1	5JZR_AB	**5JZR_A**	5JZR_B	2	0.574	Medium	TOP3	5JZR_B	3	0.064	Incorrect
T0860-D1	5FJL_ABC^a^	5FJL_BC	**5FJL_A**	1	0.82	High	5FJL_BC	TOP1	5	0.687	Medium
T0861-D1	5J5V_AD	**5J5V_A**	5J5V_D	1	0.856	High	TOP1	5J5V_D	2	0.89	High
T0862-D1	5J5V_AB	**5J5V_B**	5J5V_A	2	0.891	High	TOP3	5J5V_A	4	0.053	Incorrect
T0863-D2	5SY1_AB^b^	**5SY1_A**	5SY1_B	3	0.807	High	TOP5	5SY1_B	9	0.022	Incorrect
T0868-D1	5J4A_AB	**5J4A_A**	5J4A_B	1	0.733	Medium	TOP1	5J4A_B	9	0.508	Medium
T0869-D1	5J4A_AB	5J4A_A	**5J4A_B**	1	0.733	Medium	5J4A_A	TOP4	1	0.169	Incorrect
T0870-D1	5J5V_BC	5J5V_B	**5J5V_C**	1	0.715	Medium	5J5V_B	TOP2	1	0.326	Acceptable
T0873-D1	6DA6_ABCD^c^	6DA6_BCD	**6DA6_A**	3	0.46	Acceptable	6DA6_BCD	TOP4	4	0.567	Medium
T0878-D1	5UNB_AB	**5UNB_A**	5UNB_B	1	0.844	High	TOP5	5UNB_B	2	0.065	Incorrect
T0880	5N83_ABC^a^	**5N83_A**	5N83_BC	8	0.815	High	TOP4	5N83_BC	9	0.143	Incorrect
T0884-D1	5T87_AE	5T87_A	**5T87_E**	1	0.83	High	5T87_A	TOP1	2	0.442	Acceptable
T0885-D1	5T87_AE	**5T87_A**	5T87_E	1	0.83	High	TOP2	5T87_E	10	0.408	Acceptable
T0887-D1	6F03_AB	**6F03_A**	6F03_B	2	0.862	High	TOP2	6F03_B	4	0.072	Incorrect
T0889-D1	5JO9_ABCD^c^	**5JO9_A**	5JO9_BCD	1	0.532	Medium	TOP1	5JO9_BCD	2	0.447	Acceptable
T0893	5IDJ_AB	**5IDJ_A**	5IDJ_B	1	0.757	Medium	TOP4	5IDJ_B	3	0.079	Incorrect
T0894-D2	5HKQ_AI^b^	5HKQ_I	**5HKQ_A**	6	0.67	Medium	5HKQ_I	TOP1	1	0.444	Acceptable
T0895-D1	5HKQ_AI	5HKQ_A	**5HKQ_I**	2	0.585	Medium	5HKQ_A	TOP4	7	0.369	Acceptable
T0909-D1	5G5N_ABC^a^	**5G5N_A**	5G5N_BC	1	0.74	Medium	TOP5	5G5N_BC	6	0.408	Acceptable
T0917-D1	5YVR_AB	5YVR_B	**5YVR_A**	1	0.669	Medium	5YVR_B	TOP3	2	0.661	Medium
T0921-D1	5M2O_AB	**5M2O_A**	5M2O_B	5	0.909	High	TOP1	5M2O_B	4	0.09	Incorrect
T0922-D1	5M2O_AB	5M2O_A	**5M2O_B**	5	0.909	High	5M2O_A	TOP5	7	0.088	Incorrect
T0945-D1	**6BW6_AB**	**6BW6_A**	6BW6_B	5	0.64	Medium	TOP2	6BW6_B	7	0.405	Acceptable

a Modeled trimer chain A as one subunit and chains BC as the other.

b Modeled CASP designated D2 domain only, and D2-D2 interface.

c Modeled tetramer chain A as one subunit and chains BCD as the other.

**Table 3:** Docking of CASP12 refinement targets, best DockQ score of top 10 ClusPro Models.

CASP ID	Native PDB ID	Docking of X-ray structures					Docking of models to X-ray structures				
		protein 1 ligand	protein 2 receptor	ClusPro rank	DockQ	accuracy	protein 1 ligand	protein 2 receptor	ClusPro rank	DockQ	accuracy
TR862	5J5V_AB	**5J5V_B**	5J5V_A	2	0.891	High	TOP1	5J5V_A	1	0.044	Incorrect
TR868-D1	5J4A_AB	**5J4A_A**	5J4A_B	8	0.827	High	TOP1	5J4A_B	4	0.504	Medium
TR869	5J4A_AB	5J4A_A	**5J4A_B**	1	0.733	Medium	5J4A_A	TOP4	7	0.155	Incorrect
TR870-D1	5J5V_BC	**5J5V_C**	5J5V_B	1	0.645	Medium	TOP2	5J5V_B	2	0.161	Incorrect
TR884	5T87_AE	5T87_A	**5T87_E**	1	0.83	High	5T87_A	TOP2	7	0.432	Acceptable
TR885-D1	5T87_AE	**5T87_A**	5T87_E	3	0.73	Medium	TOP1	5T87_E	1	0.559	Medium
TR887	6F03_AB	**6F03_A**	6F03_B	2	0.862	High	TOP2	6F03_B	7	0.552	Medium
TR894	5HKQ_AI^a^	5HKQ_I	**5HKQ_A**	6	0.67	Medium	5HKQ_I	TOP4	7	0.651	Medium
TR895	5HKQ_AI	5HKQ_A	**5HKQ_I**	2	0.585	Medium	5HKQ_A	TOP5	10	0.131	Incorrect
TR909	5G5N_ABC^b^	**5G5N_A**	5G5N_BC	1	0.74	Medium	TOP1	5G5N_BC	3	0.346	Acceptable
TR917	5YVR_AB	5YVR_B	**5YVR_A**	1	0.669	Medium	5YVR_B	TOP5	2	0.639	Medium
TR921	5M2O_AB	**5M2O_A**	5M2O_B	5	0.909	High	TOP4	5M2O_B	7	0.058	Incorrect
TR922-D1	5M2O_AB	5M2O_A	**5M2O_B**	4	0.901	High	5M2O_A	TOP3	7	0.825	High
TR945	6BW6_AB	**6BW6_A**	6BW6_B	5	0.64	Medium	TOP1	6BW6_B	8	0.298	Acceptable

a Modeled 5HKQ, chain A trimmed to domain given by sequence.

b Modeled trimer chain A as the ligand and chains BC as the receptor.

As shown in Table 2, docking the X-ray structures we obtained high or medium accuracy results for almost all targets. The only exception is T0873-D1, for which the best docked structure is only acceptable accuracy. It is important that since we dock back the structures taken from the complexes, the success rate of ClusPro docking is biased towards experimental structures in bound conformation. Switching from X-ray structure of the target to its models, the quality of docking remains in the same category for three targets, gets better only for target T0873-D1, and drops into a lower category for all other targets. However, for 13 of the 23 targets the docking of models still yields acceptable or better docked structures, which means a 57% success rate. This percentage is about the same as obtained when docking separately crystallized protein structures using the ClusPro server [60]. Here we need to note that ClusPro is a “soft” rigid body docking method, which means that the scoring function allows for some level of steric overlap, but the approach may not be able to overcome large conformational changes upon protein–protein association [60].

The docking of models fails for ten of the 23 targets (T0859, T0862, T0863, T0869, T0878, T0880, T0887, T0893, T0921, and T0922). As shown in Table 4SI, five of these (T0859, T0863, T0869, T0878, and T0880) have very low GDT_TS scores, so it is not surprising that the models cannot form complexes. The models for T0893 are also of rather poor quality, with the highest GDT-TS score of 61.98. We have already discussed that target T0862 (Chain B in 5J5V) is a 3-helix bundle with flexible loops on one end that are supposed to fit into the major cavity of 5J5V_A, but due to the conformational changes in multiple loops the association is not feasible. Target T0887 (PDB ID 6F03) forms a dimer by co-folding of helical regions. Although the best model has the moderate 6F03 GDT_TS score of 57.45, it is difficult to see how the dimer can be formed without unfolding and refolding. It is not entirely clear why the dockings of the models for the heterodimer protein 5M2O (targets T0921 and T0922) are so difficult. The docking of the X-ray structures yield a high accuracy complex. The top GDT_TS values for T0921 and T0922 are 70.65 and 83.78, respectively, so the models are fairly good, and none of the models exhibit unstructured regions in the interface that would explain why no highly ranked near-native docked structures are found. We have performed the same analyses for the 14 refinement targets. The results, listed in Table 3, show limited deviation from the result for regular targets. Indeed, the accuracy level of model docking became worse for ten targets, and remained the same for four targets.

**Fig. 8:**
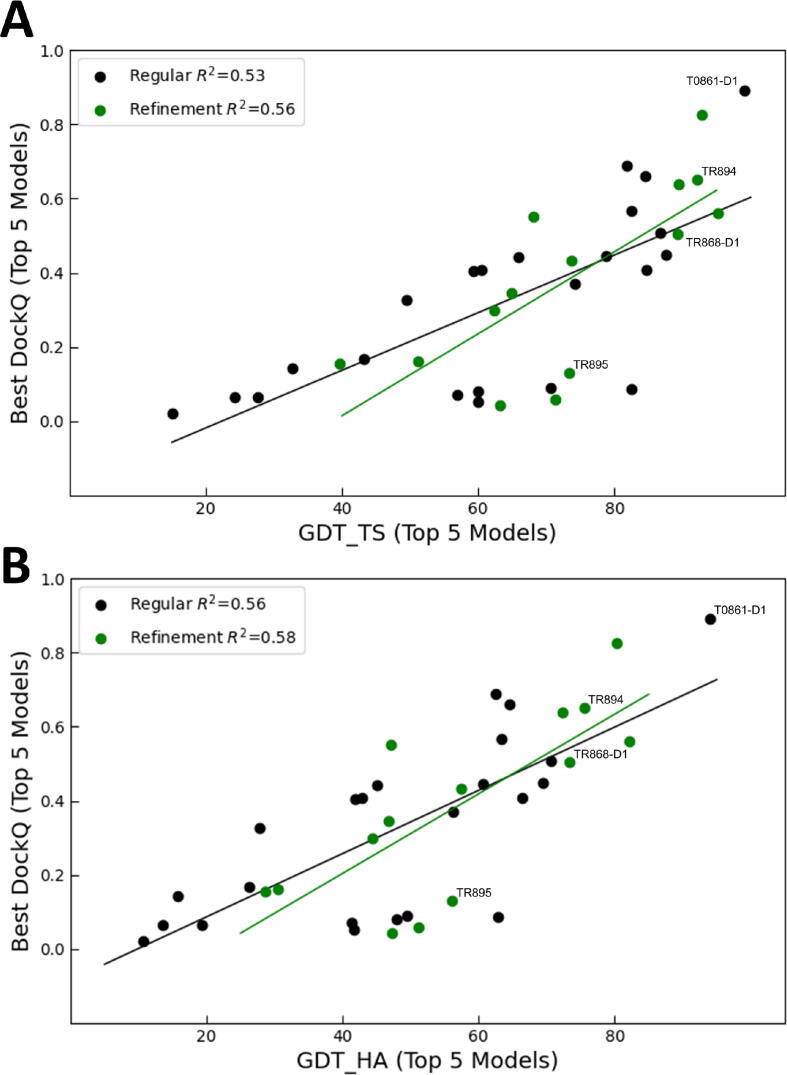
Best DockQ score within the first 10 ClusPro-produced docking models versus GDT scores. (A) DockQ score vs. GDT_TS. (B) DockQ score vs. GDT_HA. Regular targets are shown as black dots, and the linear regression is shown as a black line. Refinement targets are shown as green dots, and the linear regression is shown as a green line. (For interpretation of the references to color in this figure legend, the reader is referred to the web version of this article.)

Fig. 8A shows the relationship between the DockQ score and GDT_TS for both the regular and the refinement targets. As mentioned, for each target we dock the five best CASP models, and for each model retain the 10 best docked structures. Here we show the best DockQ score attained, the ones listed in Table 2, Table 3. The figure indicates that, on the average, refinement does not improve the docking results. The highest DockQ value is achieved for T0861, the target already discussed. The average GDT_TS for T0861 is 98.77, and as we mentioned, the protein (5J5V_A) has a well-defined large cavity that binds the C-terminal Gly-Tyr-Gly-Ile peptide tail of the partner protein, 5J5V_B. The relationship between the DockQ score and GDT_HA (Fig. 8B) shows the same structures as outliers, and since it provides essentially the same information as Fig. 8A, it will not be discussed separately.

## 4 Conclusions

Functional analysis of CASP targets in the past has heavily relied on notes from the experimentalist to determine regions of interest on the protein, or potential binding sites [7], [8], [9]. While this information can be incredibly valuable, it also limits the functional analysis of the CASP predicted structures by necessitating target-specific analyses, rather than a more generic approach. This is the case again in CASP12, where only 9 of 51 regular CASP targets were co-crystalized with a ligand. As the majority of structures in CASP12 are crystalized as apo structures, there is no one particular binding site of interest to focus on for these cases. Therefore, to compare the surface binding properties of the target proteins to the properties of their models we developed the concept of binding fingerprint, which measures the interactions of individual residues with small molecules sampling the protein surface. The fingerprints can be used to compare all binding regions on a predicted structure to the X-ray structure by calculating the Pearson correlation coefficient (PCC) between the two fingerprints. Calculating PCC values for 51 regular and 31 refinement targets has shown that the conservation of surface binding properties correlate with the accuracy of the models measured in terms of GDT_TS. Comparing different X-ray structures of the same proteins was used to establish the threshold PCC = 0.5 as the lowest correlation between the binding fingerprints of such structures. Thus, we assume that PCC > 0.5 between the X-ray structure and a model of a protein indicates good capture of surface binding properties by the model. Based on the results of our analysis we concluded that with a few exceptions, PCC ≥ 0.5 occurs only for models with GDT_TS ≥ 80. It was shown that models achieve this accuracy for a substantial fraction of CASP12 targets, but the PCC values exceed 0.5 for only about half of such targets, because the binding properties are determined by both global and local measures of accuracy. However, while high GDT_TS is generally required for PCC > 0.5, high GDT_TS or GDT_HA values do not guarantee a high PCC. Thus, the surface binding properties of a protein cannot be modeled well without high quality modeling of the fold, but the global accuracy of the backbone does not necessarily imply accurate modeling of surface properties (see Fig. 4). As shown in Fig. 5, this statement remains true when GDT_TS is replaced by side chain focused measures GDC_SC and LDDT.

The advantage of our analysis based on comparing fingerprints is that we were able to study 82 targets and thus to reach some general conclusions. In contrast, we had only nine structures with known binding sites. The analysis of these structures suggests that for the identification of binding sites capable of binding drug-sized molecules with high enough affinity it may be sufficient to reach GDT_TS values around 60, since the probes tend to cluster in large cavities (see Table 1). However, it was also shown that small conformational differences may affect the ability of the site to actually accommodate ligands, and hence generating and analyzing an ensemble of models may be necessary to estimate the level of reliability. The results of docking small ligands to the models of targets T0889 and T0893, with the average GDT_TS values of 86.69 and 60.74, suggest that substantially better models yield higher accuracy docking results. However, the smaller variations in the GDT_TS values among the models of the same target do not reflect the local conformational changes that can substantially impact the accuracy of docking. As potential caveats we note that docking of small molecules in this paper was restricted to three targets, and we used Autodock Vina [15], which accounted for the rotational degrees of freedom of the ligand, but kept the X-ray structure of the receptor extracted from the protein–ligand complex rigid, Thus, this part of our study is clearly limited, and proper analysis would require much larger sets of models for proteins that have known bound ligands, and possibly the use of other docking programs. In addition, given the fact that here we had only three examples for docking, we did not consider the quality of crystal structures as a condition for inclusion in this study.

We have also studied the ability of the models to form protein–protein complexes seen in the X-ray structures. Docking calculations were carried out for 23 regular and 14 refinement targets. Results suggest that this application is more demanding than the identification of small ligand binding sites. Rigid body programs and servers such as ClusPro [13] used here are consistently among the best performers in the CAPRI experiments for docking separately crystallized protein structures [60]. The methods allow for some steric clashes and hence can account for moderate conformational changes upon forming a complex. Both the CAPRI results and application to protein docking benchmark sets show about 60% success rates [60]. Docking models to X-ray structures of partner proteins extracted from the complexes we observe similar success rates. However, the docking almost always fails if the interface region of the modeled protein includes loops, particularly if the loops are in a convex region of the structure rather than in a well-defined cavity. In such structures loops have considerable conformational freedom. Mispredicted loops in a model may have minor impact on the GDT_TS score, but lead to steric clashes that prevent forming near-native complexes. We think that the need for improving the accuracy of loops for assembly predictions was made less urgent by the success of template based docking methods in both CASP and CAPRI, particularly because most targets have been homo-oligomers with fairly similar structures available in the PDB [61], [62], [63], [64], [65]. However, such templates will not always be available, and we emphasize here that docking protein models is still an important challenge [66].

## CRediT authorship contribution statement

**Megan Egbert:** Conceptualization, Data curation, Formal analysis, Investigation, Validation, Visualization, Writing - original draft. **Kathryn A. Porter:** Investigation. **Usman Ghani:** Investigation, Validation. **Sergei Kotelnikov:** Investigation. **Thu Nguyen:** Investigation. **Ryota Ashizawa:** Investigation, Data curation. **Dima Kozakov:** Conceptualization, Data curation, Formal analysis, Investigation, Methodology, Project administration, Resources, Software, Supervision, Funding acquisition. **Sandor Vajda:** Conceptualization, Data curation, Formal analysis, Investigation, Validation, Methodology, Project administration, Resources, Software, Supervision, Writing - original draft, Writing - review & editing, Funding acquisition.

## Declaration of Competing Interest

The authors declare that they have no known competing financial interests or personal relationships that could have appeared to influence the work reported in this paper.
