# Supplementary material for: Conservation of binding properties in protein models
Source: Comput Struct Biotechnol J. 2021 Apr 25;19:2549–66. doi: 10.1016/j.csbj.2021.04.048 (PMC8114079; doi:10.1016/j.csbj.2021.04.048)
Supplement: Supplementary data 1 [file mmc1.pdf]

Supporting Information

Conservation of binding properties in protein models

Megan Egbert[1‡], Kathryn A. Porter[1‡], Usman Ghani[1], Sergey Kotelnikov[2,3], Thu Nguyen[3],

Ryota Ashizawa[2,3], Dima Kozakov[2,3], and Sandor Vajda[1,4*]

[1]Department of Biomedical Engineering, Boston University, Boston, Massachusetts 02215, United States
[2]Department of Applied Mathematics and Statistics, Stony Brook University, Stony Brook, New York, 11794, United States
[3]Laufer Center for Physical and Quantitative Biology, Stony Brook University, Stony Brook, New York, 11794, United States
[4]Department of Chemistry, Boston University, Boston, Massachusetts 02215, United States

Table 1 SI. CASP12 regular targets with an experimentally determined structure deposited in the PDB

Target[a]	Domains	Number of Residues	PDB ID	Query Cover	Percent Identity
T0859	D1	133	5JZR_A	97%	98.46%
T0860	D1	137	5FJL_A	100%	100%
T0861	D1	323	5J5V_A	100%	99.69%
T0862	D1	239	5J5V_B	96.04%	94%
T0863	D1, D2	670	5SY1_A	100%	100%
T0864	D1	246	5D9G_A	100%	95.93%
T0865	D1	75	2N64_A	100%	100%
T0866	D1	183	5UW2_A	83%	100%
T0868	D1	161	5J4A_A	100%	100%
T0869	D1	120	5J4A_B	100%	100%
T0870	D1	138	5J5V_C	99%	98.54%
T0872	D1	91	5JMB_A	100%	97.80%
T0873	D1	501	6DA6_A	100%	100%
T0877	D1	142	5NSJ_A	100%	100%
T0878	D1	358	5UNB_A	100%	100%
T0879	D1	223	5JMU_A	100%	97.76%
T0880	D1, D2	193	5N83_A	100%	100%
T0882	D1	89	5G3Q_A	100%	100%
T0883	D1	237	6EIO_A	100%	100%
T0884	D1	75	5T87_E	100%	98.67%
T0885	D1	116	5T87_A	99%	97.39%
T0886	D1, D2	346	5FHY_A	100%	100%
T0887	D1	178	6F03_A	100%	97.75%
T0889	D1	242	5JO9_A	100%	100%
T0891	D1	130	4YMP_A	100%	100%
T0892	D1, D2	193	5NV4_A	100%	100%
T0893	D1, D2	242	5IDJ_A	100%	100%
T0894	D1, D2	324	5HKQ_A	44%	99.30%
T0895	D1	129	5HKQ_I	99%	98.44%

T0900	D1	106	5AOT_A	100%	99.06%
T0902	D1	315	3JB5_A	100%	100%
T0903	D1	382	5A7D_B	100%	100%
T0904	D1	341	5A7D_L	100%	100%
T0907	D1, D2, D3	315	7CMG_A	100%	100%
T0909	D1	340	5G5N_A	100%	96.47%
T0910	D1	345	6BDL_A	100%	99.71%
T0911	D1	445	6E9N_A	100%	100%
T0912	D1, D2, D3	624	5MQP_A	100%	100%
T0914	D1, D2	337	6CP8_A	48%	96.34%
T0915	D1	161	6CP8_C	99%	97.50%
T0917	D1	409	5YVR_A	100%	99.76%
T0918	D1, D2, D3	546	5UVN_A	72%	99.24%
T0920	D1, D2	568	5ERE_A	100%	98.42%
T0921	D1	149	5AOZ_A	97%	97.95%
T0922	D1	96	5M2O_B	100%	100%
T0928	D1	388	5TF2_A	100%	100%
T0942	D1, D2	487	6AIT_A	89%	100%
T0943	D1, D2	563	5KKP_A	100%	96.98%
T0944	D1	277	5KO9_A	100%	100%
T0945	D1	409	5LEV_A	100%	100%
T0948	D1	166	5TJ4_A	99%	98.79%

[a]All experimental structures were trimmed (if needed) to match the sequence of the target or target-domain.
[b]The PDB structure for each of the 51 targets shown here was identified by a BLAST search of the sequence against the PDB; the query coverage and percent identity are recorded for structure listed in the PDB ID column.

Table 2 SI. CASP12 refinement targets with an experimentally determined structure deposited in the PDB

Targets[a]	Domains	Number of Residues	Reference Target	PDB ID[b]	Query Cover	Percent Identity
TR520	-	321	T0920-D1	5ERE_A	100%	98.75%
TR594	-	89	T0894-D1	5HKQ_A	100%	100%
TR694	-	263	T08984 + T0895	5HKQ_A | 5HKQ_I	54% | 45%	99.30% | 98.32%
TR862	-	101	T0862-D1	5J5V_B	100%	95.79%
TR866	D1	115	T0866	5UW2_A	100%	100%
TR868	D1	116	T0868	5J4A_A	100%	100%
TR869	-	104	T0869	5J4A_B	100%	100%
TR870	D1	123	T0870	5J5V_C	100%	98.37%
TR872	-	88	T0872	5JMB_A	100%	97.73%
TR877	-	142	T0877	5NSJ_A	100%	100%
TR879	-	220	T0879	5JMU_A	100%	97.73%
TR882	-	79	T0882	5G3Q_A	100%	100%
TR884	-	72	T0884	5T87_E	100%	98.61%
TR885	D1	114	T0885	5T87_A	100%	97.37%
TR887	D9	161	T0887	6F03_A	100%	97.52%
TR891	-	119	T0891	4YMP_A	100%	100%
TR893	-	169	T0893-D2	5IDJ_A	100%	100%
TR894	-	54	T0894-D2	5HKQ_A	100%	98.15%
TR895	-	120	T0895	5HKQ_I	99%	98%
TR909	-	340	T0909	5G5N_A	100%	96.47%
TR910	-	317	T0910	6BDL_A	100%	99.68%
TR912	-	414	T0912-D1	5MQP_A	100%	99.09%
TR917	-	391	T0917	5YVR_A	100%	99.74%
TR920	-	241	T0920-D2	5ERE_A	100%	97.93%
TR921	-	138	T0921	5AOZ_A	100%	97.83%
TR922	D1	74	T0922	5M2O_B	100%	100%
TR928	-	381	T0928	5TF2_A	100%	100%
TR942	-	387	T0942	6AIT_A	99%	88.53%
TR944	-	270	T0944	5KO9_A	100%	100%
TR945	-	396	T0945	5LEV_A	100%	100%
TR948	-	161	T0948	5TJ4_A	100%	98.76%

[a]All experimental structures were trimmed (if needed) to match the sequence of the target or target-domain.
[b]The PDB structure for each of the 31 targets shown here was identified by a BLAST search of the refined sequence against the PDB; the query coverage and percent identity recorded correspond to the structure listed as the PDB ID.

Table 3 SI. Targets for Protein-Protein Docking

Regular Target	Refinement Target	PDB Complex	Oligomeric State	
			PDB	PISA
T0859-D1	--	5JZR_AB[a]	A2	-
T0860-D1	--	5FJL_ABC	A3	A3
T0861-D1	--	5J5V_AD[c]	A1B1C1	A2B2C2
T0862-D1	TR862	5J5V_AB[c]	A1B1C1	A2B2C2
T0863-D2	--	5SY1_AB[b]	A2B2	-
T0868-D1	TR868-D1	5J4A_AB	A1B1	A1B1
T0869-D1	TR869	5J4A_AB	A1B1	A1B1
T0870-D1	TR870-D1	5J5V_BC[c]	A1B1C1	A2B2C2
T0873-D1	--	6DA6_ABCD	A4	A4
T0878-D1	--	5UNB_AB	A2	A2
T0880	--	5N83_ABC	A3	A3
T0884-D1	TR884	5T87_AE	A1B1	A1B1
T0885-D1	TR885-D1	5T87_AE	A1B1	A1B1
T0887-D1	TR887	6F03_AB	A2	A2
T0889-D1	--	5JO9_ABCD	A4	A4
T0893	--	5IDJ_AB	A2	A2
T0894-D2	TR894	5HKQ_AI	A1B1	A1B1
T0895-D1	TR895	5HKQ_AI	A1B1	A1B1
T0909-D1	TR909	5G5N_ABC	A3	A3
T0917-D1	TR917	5YVR_AB	A2	A2
T0921-D1	TR921	5M2O_AB	A1B1	A1B1
T0922-D1	TR922-D1	5M2O_AB	A1B1	A1B1
T0945-D1	TR945	6BW6_AB	A2	A2

[a]5JZR_AB is an NMR structure

[b]PISA did not produce a result for 5SY1_AB

[c]5J5V has A1B1C1 as biological assembly in the PDB, but A2B2C2 according to PISA

Table 4 SI. GDT_TS, GDT_HA, and binding fingerprint PCC values for the top 5 ranked models of the CASP12 regular targets

Target	PDB ID	GDT_TS			GDT_HA			Binding Fingerprint PCC		
		Best	Avg	Stdev	Best	Avg	Stdev	Best	Avg	Stdev
T0859	5JZR_A	28.32	27.79	0.26	19.91	19.60	0.39	0.22	0.05	0.14
T0860	5FJL_A	81.80	80.51	0.99	62.69	62.10	0.83	0.56	0.42	0.11
T0861	5J5V_A	99.04	98.77	0.19	94.47	93.57	0.98	0.87	0.79	0.09
T0862-D1	5J5V_B	61.56	60.27	0.98	45.43	42.31	1.71	0.39	0.33	0.04
T0863	5SY1_A	10.22	9.86	0.23	6.79	6.63	0.11	0.08	0.07	0.02
T0864	5D9G_A	35.87	33.27	1.69	23.98	22.07	1.55	0.17	0.11	0.06
T0865	2N64_A	87.10	86.86	0.32	74.59	71.69	1.83	0.76	0.58	0.12
T0866	5UW2_A	74.13	73.87	0.16	57.17	56.69	0.63	0.54	0.37	0.18
T0868	5J4A_A	86.64	81.34	2.69	70.69	64.48	3.13	0.46	0.38	0.11
T0869	5J4A_B	52.40	45.34	3.64	31.49	27.45	2.11	0.08	0.02	0.07
T0870	5J5V_C	51.63	48.29	2.36	32.52	27.81	2.65	0.33	0.21	0.13
T0872	5JMB_A	77.84	76.88	0.87	61.65	59.09	1.92	0.43	0.34	0.06
T0873	6DA6_A	83.50	82.93	0.46	63.69	63.13	0.45	0.76	0.69	0.05
T0877	5NSJ_A	70.25	69.65	0.43	49.12	48.20	0.52	0.24	0.19	0.04
T0878	5UNB_A	26.74	25.45	0.80	15.92	13.97	1.18	0.28	0.18	0.09
T0879	5JMU_A	79.20	78.09	0.76	64.20	62.93	0.90	0.75	0.54	0.13
T0880	5N83_A	33.03	32.87	0.15	16.06	15.90	0.10	0.33	0.30	0.02
T0882	5G3Q_A	91.46	90.89	0.31	76.27	74.63	1.10	0.71	0.68	0.03
T0883	6EIO_A	89.40	89.38	0.04	77.19	76.75	0.31	0.36	0.31	0.03
T0884	5T87_E	65.84	57.18	4.35	45.07	37.61	3.75	0.45	0.23	0.13
T0885	5T87_A	87.94	83.42	3.57	73.69	66.19	5.51	0.76	0.55	0.20
T0886	5FHY_A	35.48	34.87	0.72	25.32	23.67	1.05	-0.05	-0.05	0.00
T0887	6F03_A	57.45	56.65	0.47	41.77	40.34	0.99	0.20	0.10	0.06
T0889	5JO9_A	87.55	86.69	0.62	69.36	68.92	0.43	0.80	0.70	0.09
T0891	4YMP_A	91.74	91.34	0.26	76.34	75.49	0.95	0.47	0.38	0.08
T0892	5NV4_A	42.62	39.58	1.88	26.43	25.75	0.50	0.12	0.01	0.07
T0893	5IDJ_A	61.98	60.74	0.91	49.07	47.81	0.86	0.67	0.60	0.07
T0894	5HKQ_A	59.09	55.98	2.04	38.99	35.03	2.64	0.62	0.23	0.25
T0895	5HKQ_I	75.42	74.46	0.49	56.25	55.87	0.46	0.47	0.22	0.21
T0900	5AOT_A	66.18	62.26	2.70	45.84	43.04	1.70	0.70	0.44	0.17
T0902	3JB5_A	50.75	49.80	0.52	31.25	30.67	0.45	0.22	0.20	0.02
T0903	5A7D_B	93.97	92.70	1.70	83.33	80.75	2.81	0.83	0.74	0.09
T0904	5A7D_L	36.66	36.08	0.31	29.18	27.89	0.81	0.24	0.12	0.08
T0907	7CMG_A	24.74	24.35	0.23	14.38	13.54	0.67	0.28	0.02	0.13
T0909	5G5N_A	62.01	60.95	0.59	44.97	43.26	1.16	0.53	0.33	0.14
T0910	6BDL_A	87.91	86.49	0.74	70.89	69.73	1.09	0.70	0.65	0.03
T0911	6E9N_A	65.99	65.60	0.33	44.79	44.29	0.29	0.83	0.70	0.08
T0912	5MQP_A	49.08	48.35	0.37	35.18	34.29	0.62	0.07	0.04	0.02

T0914	6CP8_A	19.38	19.17	0.14	12.35	11.94	0.23	0.01	-0.01	0.01
T0915	6CP8_C	52.44	51.75	0.60	30.68	30.20	0.58	0.57	0.28	0.16
T0917	5YVR_A	84.18	83.04	0.92	64.02	63.00	0.94	0.68	0.64	0.03
T0918	5UVN_A	28.58	25.19	2.09	13.11	12.15	0.58	0.40	0.27	0.10
T0920	5ERE_A	50.42	48.22	1.14	34.82	32.42	1.82	0.23	0.15	0.06
T0921	5AOZ_A	70.65	69.96	0.58	50.18	48.44	1.21	0.51	0.09	0.21
T0922	5M2O_B	83.78	83.18	0.50	68.58	66.83	2.17	0.51	0.31	0.17
T0928	5TF2_A	63.27	62.99	0.24	43.70	43.52	0.19	0.85	0.79	0.05
T0942	6AIT_A	54.52	53.13	0.81	35.08	34.38	0.44	0.46	0.10	0.21
T0943	5KKP_A	60.61	59.24	1.26	43.08	41.24	1.54	0.73	0.56	0.11
T0944	5KO9_A	76.28	76.09	0.18	59.98	58.91	0.64	0.51	0.36	0.11
T0945	5LEV_A	59.73	59.04	0.46	42.00	41.39	0.61	0.72	0.43	0.19
T0948	5TJ4_A	76.68	76.11	0.42	60.74	59.03	1.30	0.43	0.25	0.20

	GDT_TS	GDT_HA	Binding Fingerprint PCC
**Average**	61.31	45.42	0.33
**St. Deviation**	22.39	21.05	0.24

Table 5 SI. GDT_TS, GDT_HA, and binding fingerprint PCC values for the top 5 ranked models of the CASP12 refinement targets

Target	PDB ID	GDT_TS			GDT_HA			Binding Fingerprint PCC		
		Best	Avg	Stdev	Best	Avg	Stdev	Best	Avg	Stdev
TR520	5ERE_A	80.69	80.27	0.35	60.12	59.61	0.29	0.53	0.45	0.05
TR594	5HKQ_A	73.60	70.39	2.07	53.65	51.46	1.50	0.65	0.42	0.13
TR694	5HKQ_A	53.42	49.64	2.02	32.70	30.19	1.46	0.14	0.06	0.05
TR862	5J5V_B	63.17	62.47	0.69	47.31	45.27	1.33	0.43	0.35	0.05
TR866	5UW2_A	82.83	79.48	2.11	66.96	63.30	2.30	0.68	0.45	0.16
TR868	5J4A_A	85.13	82.76	1.23	68.75	64.96	2.25	0.87	0.78	0.11
TR869	5J4A_B	47.12	41.97	2.86	37.02	30.77	3.48	0.46	0.22	0.17
TR870	5J5V_C	48.78	46.14	1.92	27.84	27.52	0.28	0.62	0.20	0.23
TR872	5JMB_A	77.27	76.87	0.34	60.23	58.98	1.12	0.25	0.19	0.05
TR877	5NSJ_A	72.53	72.32	0.17	51.94	51.55	0.53	0.28	0.19	0.06
TR879	5JMU_A	79.89	79.59	0.21	64.66	64.50	0.14	0.67	0.57	0.10
TR882	5G3Q_A	92.72	91.58	0.59	78.17	76.39	1.52	0.73	0.57	0.14
TR884	5T87_E	76.06	72.89	1.80	59.16	54.86	2.87	0.43	0.26	0.14
TR885	5T87_A	90.79	90.22	0.45	77.41	76.49	0.65	0.83	0.62	0.12
TR887	6F03_A	72.05	65.19	4.34	51.09	48.14	2.32	0.79	0.42	0.33
TR891	4YMP_A	92.41	91.96	0.24	77.68	76.70	0.75	0.68	0.51	0.09
TR893	5IDJ_A	88.76	88.43	0.17	73.22	71.78	0.86	0.88	0.80	0.09
TR894	5HKQ_A	95.83	92.59	1.80	80.56	76.85	2.59	0.95	0.92	0.02
TR895	5HKQ_I	77.29	74.16	1.57	61.88	57.46	2.30	0.54	0.44	0.06
TR909	5G5N_A	64.79	62.58	1.11	46.70	45.21	0.86	0.37	0.19	0.11
TR910	6BDL_A	86.64	86.60	0.04	72.69	72.05	0.39	0.86	0.57	0.17
TR912	5MQP_A	67.94	67.50	0.33	53.68	51.70	1.53	0.22	0.16	0.04
TR917	5YVR_A	89.96	89.59	0.25	73.21	72.75	0.38	0.76	0.73	0.02
TR920	5ERE_A	82.76	82.03	0.41	64.61	63.54	0.59	0.27	0.11	0.09
TR921	5AOZ_A	73.55	71.85	0.87	54.16	52.35	1.13	0.31	0.17	0.14
TR922	5M2O_B	88.51	86.96	0.94	74.33	72.70	1.34	0.33	0.28	0.06
TR928	5TF2_A	69.80	68.89	0.54	50.73	49.80	1.11	0.85	0.75	0.09
TR942	6AIT_A	64.73	60.83	2.03	50.71	44.76	3.42	0.65	0.31	0.17
TR944	5KO9_A	78.16	76.52	0.84	59.59	58.98	0.33	0.55	0.44	0.08
TR945	5LEV_A	62.40	61.64	0.45	46.06	44.29	1.00	0.53	0.44	0.08
TR948	5TJ4_A	81.71	80.80	0.72	68.62	66.51	1.78	-0.01	-0.03	0.01

	GDT_TS	GDT_HA	Binding Fingerprint PCC
**Average**	74.35	57.46	0.40
**St. Deviation**	13.50	14.01	0.27

Table 6 SI. FTMap PCC between close homologs in the PDB for some CASP12 targets

Target	Sequence Identity	Casp12 PDB Structure		Homologous PDB Structure			
		PDB ID	PDB Sequence Length	PDB ID	PDB Sequence Length	PCC[a]	GDT_TS
T0859_homol	88.64	5JZR_A	131	5FS4_A	118	0.76	66.10
T0861_homol	99.05	5J5V_A	312	1OAS_A	315	0.83	98.02
T0861_homol	96.17	5J5V_A	312	1FCJ_A	302	0.68	92.80
T0861_homol	96.89	5J5V_A	312	1D6S_A	322	0.89	89.36
T0861_homol	100	5J5V_A	312	5J43_A	312	0.98	99.92
T0862_homol	92.08	5J5V_B	93	5J43_B	101	0.58	83.66
T0864_homol	84.49	5D9G_A	236	5W0W_B	216	0.64	92.01
T0864_homol	85.71	5D9G_A	236	5W0X_A	206	0.74	94.42
T0866_homol	97.39	5UW2_A	112	6XBD_A	115	0.77	95.44
T0866_homol	91.96	5UW2_A	112	6ZY9_A	103	0.42	91.99
T0866_homol	97.39	5UW2_A	112	7CGN_G	115	0.57	80.87
T0866_homol	100	5UW2_A	112	6ZY2_A	112	0.29	85.05
T0866_homol	98.21	5UW2_A	112	6ZY3_A	110	0.77	90.00
T0866_homol	97.39	5UW2_A	112	7CH0_G	115	0.64	94.13
T0866_homol	97.39	5UW2_A	112	7CGE_G	115	0.86	93.70
T0866_homol	91.96	5UW2_A	112	6ZY4_A	103	0.82	93.20
T0877_homol	97.26	5NSJ_A	142	6HJ4_A	146	0.55	91.27
T0877_homol	95.95	5NSJ_A	142	6HJ5_A	148	0.57	89.87
T0878_homol	99.43	5UNB_A	348	5I3E_A	346	0.84	99.78
T0880_homol	99.48	5N83_A	194	5NC1_A	193	0.87	96.89
T0880_homol	100	5N83_A	194	5NBH_A	194	0.90	97.81
T0880_homol	100	5N83_A	194	5N8D_A	194	0.93	98.33
T0892_homol	100	5NV4_A	193	5MZO_A	193	0.98	99.87
T0892_homol	100	5NV4_A	193	6TRT_A	193	0.96	99.87
T0892_homol	100	5NV4_A	193	6TS2_A	193	0.83	97.02
T0892_homol	100	5NV4_A	193	5N2J_A	193	0.53	100.00
T0892_homol	100	5NV4_A	193	5MU1_A	193	0.98	100.00
T0892_homol	100	5NV4_A	193	6TRF_A	193	0.95	99.74
T0900_homol	92.16	5AOT_A	102	5FU3_A	94	0.83	93.62
T0900_homol	98.04	5AOT_A	102	5AOS_A	100	0.99	100.00
T0900_homol	90.2	5AOT_A	102	5FU4_A	92	0.85	96.47
T0900_homol	95.1	5AOT_A	102	5FU2_A	97	0.88	98.71
T0903_homol	93.22	5A7D_B	354	4A1S_A	330	0.86	88.11
T0907_homol	96.69	7CMG_A	302	6EY5_A	292	0.71	59.93
T0909_homol	100	5G5N_A	338	5G5O_A	338	0.96	99.70
T0910_homol	99.7	6BDL_A	334	6BG2_A	335	0.75	92.16

T0910_homol	100	6BDL_A	334	6C0T_A	334	0.85	97.90
T0911_homol	94.66	6E9N_A	409	6E9O_A	393	0.62	64.50
T0914_homol	95.71	6CP8_A	157	6VEK_A	162	0.61	86.57
T0915_homol	96.2	6CP8_C	157	6VEK_I	153	0.89	98.86
T0917_homol	100	5YVR_A	403	5YVS_A	403	0.99	100.00
T0917_homol	100	5YVR_A	403	5YVM_A	403	0.98	100.00
T0921_homol	95.04	5AOZ_A	138	5M2O_A	137	0.85	97.63
T0944_homol	99.21	5KO9_A	253	6OOV_A	251	0.82	98.00
T0944_homol	97.3	5KO9_A	253	6OEA_A	258	0.76	95.54
T0944_homol	97.3	5KO9_A	253	6OE7_A	258	0.80	95.54
T0945_homol	99.21	5LEV_A	375	6FM9_A	378	0.99	99.21
T0945_homol	97.91	5LEV_A	375	5O5E_A	381	0.94	96.79
T0945_homol	98.16	5LEV_A	375	6BW5_A	378	0.78	94.97
T0945_homol	96.04	5LEV_A	375	6BW6_A	368	0.79	92.46
T0945_homol	98.94	5LEV_A	375	6FWZ_A	373	0.96	98.53
					**Average**	**0.80**	**93.26**
					**St. Deviation**	**0.16**	**8.85**

[a]Pearson correlation coefficient between the binding fingerprints of the structure in the Casp12 PDB ID column and Homologous PDB ID.

[b] GDT_TS of the homolog relative to the CASP12 PDB

Table 7 SI. GDC_SC, LDDT, and binding fingerprint PCC values for the top 5 ranked models of the CASP12 regular targets

		GDC_SC			LDDT			Binding Fingerprint PCC		
**Target**	**PDB ID**	**Best**	**Avg**	**Stdev**	**Best**	**Avg**	**Stdev**	**Best**	**Avg**	**Stdev**
T0859	5JZR_A	7.44	5.98	1.52	0.34	0.33	0.02	0.22	0.05	0.14
T0860	5FJL_A	43.52	36.48	3.94	0.72	0.69	0.02	0.56	0.42	0.11
T0861	5J5V_A	70.53	69.12	0.78	0.93	0.91	0.01	0.87	0.79	0.09
T0862-D1	5J5V_B	22.71	17.36	3.51	0.54	0.52	0.01	0.39	0.33	0.04
T0863	5SY1_A	3.48	3.04	0.25	0.33	0.32	0.01	0.08	0.07	0.02
T0864	5D9G_A	26.81	17.98	7.00	0.40	0.26	0.11	0.17	0.11	0.06
T0865	2N64_A	37.26	34.47	2.46	0.81	0.80	0.01	0.76	0.58	0.12
T0866	5UW2_A	39.53	35.88	2.75	0.72	0.70	0.01	0.54	0.37	0.18
T0868	5J4A_A	48.12	45.72	1.35	0.75	0.73	0.01	0.46	0.38	0.11
T0869	5J4A_B	9.31	8.72	0.65	0.40	0.38	0.03	0.08	0.02	0.07
T0870	5J5V_C	16.33	11.47	2.90	0.48	0.45	0.02	0.33	0.21	0.13
T0872	5JMB_A	38.46	34.77	2.90	0.66	0.64	0.01	0.43	0.34	0.06
T0873	6DA6_A	45.87	44.03	1.09	0.77	0.76	0.01	0.76	0.69	0.05
T0877	5NSJ_A	29.97	27.98	1.25	0.62	0.61	0.01	0.24	0.19	0.04
T0878	5UNB_A	7.38	6.89	0.57	0.26	0.24	0.02	0.28	0.18	0.09
T0879	5JMU_A	41.06	38.88	1.93	0.70	0.65	0.03	0.75	0.54	0.13
T0880	5N83_A	7.59	7.16	0.32	0.37	0.36	0.00	0.33	0.30	0.02
T0882	5G3Q_A	48.40	46.10	1.78	0.77	0.75	0.03	0.71	0.68	0.03
T0883	6EIO_A	55.30	48.95	4.37	0.79	0.78	0.01	0.36	0.31	0.03
T0884	5T87_E	21.49	15.94	3.13	0.53	0.45	0.05	0.45	0.23	0.13
T0885	5T87_A	45.77	39.70	3.59	0.75	0.71	0.02	0.76	0.55	0.20
T0886	5FHY_A	17.44	15.98	0.96	0.53	0.52	0.00	-0.05	-0.05	0.00
T0887	6F03_A	26.63	25.09	0.97	0.74	0.73	0.01	0.20	0.10	0.06
T0889	5JO9_A	42.61	40.83	2.12	0.74	0.72	0.02	0.80	0.70	0.09
T0891	4YMP_A	48.51	43.76	4.93	0.76	0.74	0.02	0.47	0.38	0.08
T0892	5NV4_A	12.30	11.19	0.76	0.45	0.42	0.03	0.12	0.01	0.07
T0893	5IDJ_A	32.59	29.05	3.66	0.67	0.64	0.02	0.67	0.60	0.07
T0894	5HKQ_A	21.22	16.52	4.16	0.55	0.46	0.08	0.62	0.23	0.25
T0895	5HKQ_I	36.22	33.83	2.03	0.65	0.64	0.01	0.47	0.22	0.21
T0900	5AOT_A	23.20	19.94	2.67	0.52	0.48	0.03	0.70	0.44	0.17
T0902	3JB5_A	19.84	18.64	0.61	0.53	0.52	0.01	0.22	0.20	0.02
T0903	5A7D_B	56.83	55.58	1.16	0.89	0.87	0.01	0.83	0.74	0.09
T0904	5A7D_L	27.37	21.14	4.60	0.52	0.43	0.09	0.24	0.12	0.08
T0907	7CMG_A	8.04	5.87	1.21	0.53	0.50	0.04	0.28	0.02	0.13
T0909	5G5N_A	29.47	25.96	2.45	0.55	0.53	0.01	0.53	0.33	0.14
T0910	6BDL_A	56.23	54.02	1.99	0.81	0.81	0.01	0.70	0.65	0.03
T0911	6E9N_A	26.75	24.69	1.69	0.63	0.59	0.03	0.83	0.70	0.08
T0912	5MQP_A	20.49	18.02	2.23	0.44	0.43	0.01	0.07	0.04	0.02
T0914	6CP8_A	6.11	5.99	0.08	0.39	0.39	0.00	0.01	-0.01	0.01

T0915	6CP8_C	11.17	10.07	0.62	0.53	0.51	0.01	0.57	0.28	0.16
T0917	5YVR_A	44.91	44.01	0.74	0.76	0.75	0.01	0.68	0.64	0.03
T0918	5UVN_A	7.52	5.85	0.98	0.46	0.45	0.01	0.40	0.27	0.10
T0920	5ERE_A	22.56	18.62	2.62	0.67	0.64	0.02	0.23	0.15	0.06
T0921	5AOZ_A	30.14	28.75	1.20	0.60	0.59	0.01	0.51	0.09	0.21
T0922	5M2O_B	43.32	41.04	3.11	0.75	0.73	0.01	0.51	0.31	0.17
T0928	5TF2_A	25.99	24.72	2.12	0.56	0.53	0.03	0.85	0.79	0.05
T0942	6AIT_A	17.13	15.37	1.03	0.59	0.59	0.01	0.46	0.10	0.21
T0943	5KKP_A	33.11	31.34	1.48	0.64	0.63	0.01	0.73	0.56	0.11
T0944	5KO9_A	40.53	39.63	1.00	0.70	0.68	0.02	0.51	0.36	0.11
T0945	5LEV_A	31.93	30.04	1.18	0.61	0.61	0.00	0.72	0.43	0.19
T0948	5TJ4_A	39.41	35.06	2.25	0.68	0.62	0.06	0.43	0.25	0.20

	GDT_TS	GDT_HA	Binding Fingerprint PCC
**Average**	27.20	0.58	0.33
**St. Deviation**	15.43	0.16	0.24

Table 8 SI. GDC_SC, LDDT and binding fingerprint PCC values for the top 5 ranked models of the CASP12 refinement targets

Target	PDB ID	GDC_SC			LDDT			Binding Fingerprint PCC		
		Best	Avg	Stdev	Best	Avg	Stdev	Best	Avg	Stdev
TR520	5ERE_A	39.73	39.04	0.72	0.72	0.71	0.01	0.53	0.45	0.05
TR594	5HKQ_A	31.62	28.73	2.63	0.63	0.60	0.02	0.65	0.42	0.13
TR694	5HKQ_A	20.09	16.26	3.70	0.53	0.50	0.02	0.14	0.06	0.05
TR862	5J5V_B	21.06	14.41	4.41	0.54	0.53	0.02	0.43	0.35	0.05
TR866	5UW2_A	43.67	40.80	2.49	0.75	0.73	0.02	0.68	0.45	0.16
TR868	5J4A_A	47.39	43.56	3.50	0.74	0.73	0.02	0.87	0.78	0.11
TR869	5J4A_B	17.50	12.68	3.00	0.42	0.39	0.01	0.46	0.22	0.17
TR870	5J5V_C	13.72	11.84	1.23	0.48	0.46	0.02	0.62	0.20	0.23
TR872	5JMB_A	42.01	36.15	5.87	0.67	0.64	0.03	0.25	0.19	0.05
TR877	5NSJ_A	32.45	31.40	0.79	0.64	0.64	0.00	0.28	0.19	0.06
TR879	5JMU_A	43.80	42.48	2.15	0.71	0.70	0.01	0.67	0.57	0.10
TR882	5G3Q_A	50.15	48.76	0.82	0.80	0.79	0.01	0.73	0.57	0.14
TR884	5T87_E	29.03	27.50	1.00	0.60	0.58	0.02	0.43	0.26	0.14
TR885	5T87_A	50.34	48.12	1.80	0.79	0.78	0.01	0.83	0.62	0.12
TR887	6F03_A	32.46	28.96	2.12	0.81	0.78	0.02	0.79	0.42	0.33
TR891	4YMP_A	47.45	44.15	2.29	0.75	0.74	0.02	0.68	0.50	0.09
TR893	5IDJ_A	49.87	47.82	1.61	0.78	0.76	0.01	0.88	0.80	0.09
TR894	5HKQ_A	45.59	43.49	1.61	0.80	0.77	0.02	0.95	0.92	0.02
TR895	5HKQ_I	39.60	35.53	2.91	0.67	0.65	0.01	0.54	0.44	0.06
TR909	5G5N_A	28.04	26.11	1.14	0.55	0.53	0.01	0.37	0.19	0.11
TR910	6BDL_A	59.73	58.83	1.51	0.81	0.81	0.01	0.86	0.57	0.17
TR912	5MQP_A	36.67	33.34	1.96	0.59	0.58	0.01	0.22	0.16	0.04
TR917	5YVR_A	53.78	52.66	1.13	0.81	0.80	0.01	0.76	0.73	0.02
TR920	5ERE_A	47.81	42.53	2.71	0.70	0.68	0.01	0.27	0.11	0.09
TR921	5AOZ_A	32.40	30.22	1.89	0.60	0.60	0.00	0.31	0.17	0.14
TR922	5M2O_B	52.70	49.32	2.21	0.78	0.75	0.02	0.33	0.28	0.06
TR928	5TF2_A	35.64	33.90	1.09	0.63	0.62	0.01	0.85	0.75	0.09
TR942	6AIT_A	31.73	28.04	3.33	0.69	0.67	0.02	0.65	0.31	0.17
TR944	5KO9_A	45.45	42.81	1.55	0.70	0.68	0.01	0.55	0.44	0.08
TR945	5LEV_A	31.97	29.81	1.59	0.61	0.60	0.01	0.53	0.44	0.08
TR948	5TJ4_A	43.73	42.67	0.94	0.72	0.69	0.02	-0.01	-0.03	0.01
		GDC_SC			LDDT			Binding Fingerprint PCC		
Average		35.87			0.66			0.41		
St. Deviation		11.88			0.11			0.24		

Table 9 SI. FTMap Identified Binding Site Residues for known ligand-binding sites.

Target	PDB ID	Ligand	BS Rank | # Probes	PDB Residues within 5 A of Binding Site
T0861	5J43_A	LLP	1 | 44	38_SER, 40_SER, 41_VAL, 43_CYS, 44_ARG, 45_ILE, 46_GLY, 71_GLY, 72_ASN, 73_THR, 74_GLY, 76_ALA, 143_GLN, 149_ASN, 153_HIS, 174_ALA, 175_GLY, 176_VAL, 177_GLY, 178_THR, 179_GLY, 180_GLY, 181_THR, 182_LEU, 183_THR, 229_GLY, 230_ILE, 273_SER, 277_ALA, 298_ILE, 299_LEU, 300_PRO
T0863	5SY1_A	CLR	- | 0	--
T0873	6DA6_A	FMN*	1 | 36	153_ILE, 164_ASN, 165_TRP, 166_ALA, 167_ILE, 168_ALA, 181_THR, 182_PHE, 183_ILE, 186_GLN, 187_HIS, 188_LEU, 189_GLY, 217_VAL, 218_ALA, 219_SER, 220_MET, 221_PRO, 222_LEU, 223_PRO, 226_ILE, 228_GLU, 321_TRP, 325_THR, 386_ARG
T0879	5JMU_A	ZN	2 | 22	297_ASP, 298_ASP, 346_HIS, 350_HIS, 352_TYR, 385_ARG, 386_PHE, 387_PRO, 388_GLY, 389_GLY, 420_TRP, 427_ALA, 454_LEU, 456_HIS, 458_ILE, 459_LYS
T0889	5JO9_A	SOR	1 | 43	19_ILE, 88_ASN, 90_GLY, 91_THR, 92_TYR, 112_ASN, 138_THR, 139_SER, 140_SER, 141_LEU, 142_ALA, 143_ALA, 145_PHE, 147_THR, 149_TRP, 150_GLU, 153_TYR, 157_LYS, 182_SER, 183_PRO, 184_GLY, 185_PRO, 186_VAL, 187_VAL, 188_SER, 189_ALA, 190_LEU, 191_LEU, 193_ASP, 194_TRP, 208_LEU, 240_PHE, 242_LEU
T0891	4YMP_A	HEM	1 | 41	18_TYR, 19_LYS, 20_ASP, 26_SER, 27_TYR, 28_ALA, 31_TYR, 52_ASP, 55_PHE, 56_PHE, 103_MET, 105_ILE, 107_ILE, 110_LEU, 112_TYR, 116_PHE, 117_LYS, 118_ILE
T0893	5IDJ_A	ADP	1 | 51	431_LEU, 434_ASN, 435_ALA, 436_ARG, 437_ASP, 438_ALA, 479_ASP, 481_GLY, 482_PRO, 483_GLY, 484_ILE, 485_PRO, 488_VAL, 491_LYS, 492_ILE, 493_PHE, 494_ASP, 495_PRO, 496_PHE, 497_PHE, 498_THR, 499_THR, 500_LYS, 504_GLU, 505_GLY, 506_THR, 507_GLY, 508_LEU, 509_GLY, 510_LEU, 511_ALA, 533_GLY, 534_ALA, 536_PHE
T0910	6BDL_A	ANP*	1 | 38	366_LEU, 367_GLY, 368_VAL, 369_GLY, 371_PHE, 374_VAL, 388_ALA, 390_LYS, 409_GLU, 422_VAL, 423_ARG, 438_MET, 439_GLU, 440_ALA, 441_CYS, 445_GLU, 447_TRP, 486_LYS, 488_GLU, 489_ASN, 490_LEU, 491_ILE, 501_VAL, 502_ASP, 503_PHE, 649_PHE, 652_PHE

T0911	6E9N_A	GCO	1 | 84	44_TYR, 47_ARG, 75_PHE, 76_ALA, 77_TRP, 79_TYR, 80_THR, 83_GLN, 134_ALA, 135_PRO, 136_ALA, 137_PHE, 138_PRO, 141_ASN, 157_VAL, 158_GLY, 161_THR, 162_SER, 164_GLN, 165_PHE, 264_GLN, 267_VAL, 268_ALA, 271_LEU, 272_TRP, 304_ALA, 308_VAL, 363_PHE, 366_ASN, 367_GLY, 370_SER, 371_ILE, 372_THR, 373_TRP, 374_SER, 375_LEU, 389_GLY, 390_GLY, 392_PHE, 393_ASN, 394_PHE, 396_GLY, 397_GLY, 398_LEU, 399_GLY, 400_GLY, 401_ILE

*Ligand is not co-crystalized with PDB ID; binding site determined based on residues identified in Altman et. al.

Table 10 SI. Docking of SOR to target T0889 (PDB ID 5JO9_A) and models

TARGET	GDT_TS	BEST RMSD[a]	TOP1 RMSD[b]	TOP5 RMSD[c]	Binding Fingerprint PCC
Crystal	100	2.04	2.21	2.04	1
T0889TS405_3	87.55	2.48	4.43	2.48	0.79
T0889TS405_5	87.34	2.38	4.41	2.38	0.80
T0889TS250_2	86.19	2	3.65	2	0.60
T0889TS250_5	86.19	2.55	3.28	2.55	0.68
T0889TS016_1	86.19	3.79	5.37	4.4	0.60
T0889TS250_4	86.09	3.26	5.59	4.34	0.54
T0889TS250_3	85.98	2.89	3.04	2.89	0.53
T0889TS250_1	85.98	2.44	3.06	2.69	0.61
T0889TS183_1	85.67	2.37	6.44	5.1	0.55
T0889TS275_5	85.46	2.38	5.23	2.38	0.67
T0889TS166_1	85.46	2.29	6.77	3.81	0.77
T0889TS359_4	85.15	5	5.39	5	0.79
T0889TS359_5	85.15	4.59	5.4	4.98	0.79
T0889TS405_2	85.04	1.66	6.49	2.17	0.70
T0889TS405_1	85.04	2.07	6.48	2.21	0.70
T0889TS405_4	84.94	1.6	2.35	2.35	0.70
T0889TS479_1	84.94	4.21	7.04	4.21	0.75
T0889TS452_1	84.94	1.95	2.41	2.41	0.76
T0889TS026_2	84.73	5.27	5.92	5.27	0.69
T0889TS380_1	84.73	5.27	5.93	5.27	0.78
T0889TS275_2	84.73	2.46	3.52	2.46	0.74
T0889TS380_2	84.73	4.77	5.92	5.28	0.74
T0889TS359_1	84.73	2.18	5.32	5.32	0.74
T0889TS026_1	84.73	4.99	5.92	5.57	0.74
T0889TS275_3	83.68	1.52	2.43	2.43	0.74
T0889TS275_4	83.68	3.48	4.58	3.78	0.74
T0889TS275_1	83.68	2.1	3.92	2.1	0.69
T0889TS005_2	83.58	1.94	1.94	1.94	0.74
T0889TS444_2	83.16	5.25	6.71	5.25	0.72
T0889TS005_5	83.16	2	2.18	2.07	0.65
LigTBM		2.04	2.04	2.04	
**Average**	**85.09**	**3.04**	**4.70**	**3.50**	**0.70**
**St. Deviation**	**1.07**	**1.25**	**1.56**	**1.31**	**0.08**

[a]Lowest RMSD of the docked ligand among all structures by Autodock Vina

[b]RMSD of the lowest energy (top 1) structure by Autodock Vina

[c]Best RMSD among the 5 lowest energy (top 5) structures by Autodock Vina

Table 11 SI. Docking of ADP to target T0893 (PDB ID 5IDJ_A) and models

**TARGET**	**GDT_TS**	**BEST RMSD[a]**	**TOP1 RMSD[b]**	**TOP5 RMSD[c]**	**Binding Fingerprint PCC**
Crystal	100	1.89	1.89	1.89	1
T0893TS220_5	61.98	2.48	2.48	2.48	0.67
T0893TS005_1	61.47	3.02	7.05	5.71	0.53
T0893TS220_3	60.74	3.79	5.32	3.79	0.66
T0893TS183_1	60.02	4.04	4.14	4.14	0.49
T0893TS479_1	59.5	4.13	5.2	4.13	0.64
T0893TS405_4	59.3	3.38	7.46	3.38	0.27
T0893TS479_4	59.09	3.99	4.23	4.09	0.53
T0893TS220_4	58.88	9.02	10.5	9.28	0.43
T0893TS183_3	58.78	3.57	4.53	4.25	0.55
T0893TS005_3	58.78	4.17	5.97	5.97	0.60
T0893TS313_4	57.85	3.39	3.89	3.49	0.34
T0893TS313_1	57.65	3.62	3.88	3.88	0.48
T0893TS313_2	57.54	2.82	2.82	2.82	0.55
T0893TS313_5	57.54	3.28	3.28	3.28	0.24
T0893TS405_3	57.54	3.25	3.45	3.45	0.48
T0893TS313_3	57.44	3.62	7.68	3.62	0.32
T0893TS183_2	57.23	6.93	7.96	6.93	0.42
T0893TS479_5	57.13	4.32	6.53	6.53	0.45
T0893TS183_5	57.13	4.51	7.44	4.51	0.26
T0893TS236_4	57.02	5.16	5.27	5.16	0.70
T0893TS119_1	56.92	1.84	7.56	1.84	0.45
T0893TS349_1	56.92	1.91	1.91	1.91	0.40
T0893TS467_1	56.72	4.82	7.78	4.82	0.52
T0893TS430_5	56.61	2.07	2.07	2.07	0.47
T0893TS479_2	56.61	7.31	7.57	7.31	0.66
T0893TS425_4	56.51	3.4	4.67	3.4	0.57
T0893TS287_4	56.51	6.85	7.07	6.86	0.51
T0893TS405_5	56.41	4.75	5.07	4.91	0.55
T0893TS005_2	56.3	3.44	4.1	3.44	0.52
T0893TS430_4	56.2	null	null	null	0.51
LigTBM		0.70	1.45	0.80	
**Average**	**58.00**	**4.10**	**5.41**	**4.39**	**0.49**
**St. Deviation**	**1.54**	**1.62**	**2.08**	**1.73**	**0.12**

[a]Lowest RMSD of the docked ligand among all structures by Autodock Vina

[b]RMSD of the lowest energy (top 1) structure by Autodock Vina

[c]Best RMSD among the 5 lowest energy (top 5) structures by Autodock Vina

Table 12 SI. Docking of GCO to target T0911 (PDB ID 6E9N_A) and models

TARGET	GDT_TS	BEST RMSD	TOP1 RMSD	TOP5 RMSD	Binding Fingerprint PCC
Crystal	100	5.29	6.69	5.29	1
T0911TS287_2	65.99	5.06	6.14	5.06	0.83
T0911TS320_3	65.99	5.07	6.15	5.7	0.83
T0911TS042_4	65.93	5.1	5.95	5.1	0.72
T0911TS411_2	65.62	6.75	7.51	6.87	0.72
T0911TS220_4	65.26	6.07	7.25	6.23	0.62
T0911TS004_4	65.2	5.37	6.4	5.37	0.59
T0911TS303_2	64.83	6.55	7.29	6.55	0.52
T0911TS220_5	64.71	6.16	7.25	6.24	0.40
T0911TS324_3	64.15	6.05	6.09	6.05	0.27
T0911TS324_5	63.97	6.14	6.46	6.14	0.42
T0911TS324_1	63.91	6.13	6.13	6.13	0.71
T0911TS324_4	63.91	5.96	5.96	5.96	0.42
T0911TS004_5	63.91	6.69	7.2	7.2	0.39
T0911TS393_2	63.79	6.31	7.38	6.31	0.52
T0911TS011_2	63.54	6.76	6.76	6.76	0.37
T0911TS011_5	63.36	5.89	7.19	6.1	0.77
T0911TS236_4	63.36	4.55	5.81	4.57	0.45
T0911TS324_2	63.3	5.84	6.38	5.84	0.63
T0911TS411_1	63.3	6.48	7.23	6.74	0.41
T0911TS252_1	63.23	5.62	6.83	5.62	0.49
T0911TS450_4	63.11	6.49	6.69	6.49	-0.04
T0911TS450_1	62.93	7.47	7.79	7.47	0.19
T0911TS439_2	62.81	6.94	7.43	6.94	0.38
T0911TS486_2	62.74	5.22	6.54	5.22	0.68
T0911TS252_3	62.62	3.67	3.67	3.67	0.67
T0911TS011_3	62.56	5.82	6.19	5.82	0.21
T0911TS450_3	62.5	6.69	6.71	6.69	-0.05
T0911TS325_2	62.44	6.33	6.36	6.36	0.55
T0911TS393_3	62.32	6.2	7.27	6.2	0.16
T0911TS011_1	62.32	6.3	7.05	6.3	0.51
LigTBM		7.39	7.39	7.39	
**Average**	**63.79**	**5.99**	**6.64**	**6.06**	**0.48**
**St. Deviation**	**1.14**	**0.77**	**0.77**	**0.78**	**0.23**

[a]Lowest RMSD of the docked ligand among all structures by Autodock Vina

[b]RMSD of the lowest energy (top 1) structure by Autodock Vina

[c]Best RMSD among the 5 lowest energy (top 5) structures by Autodock Vina

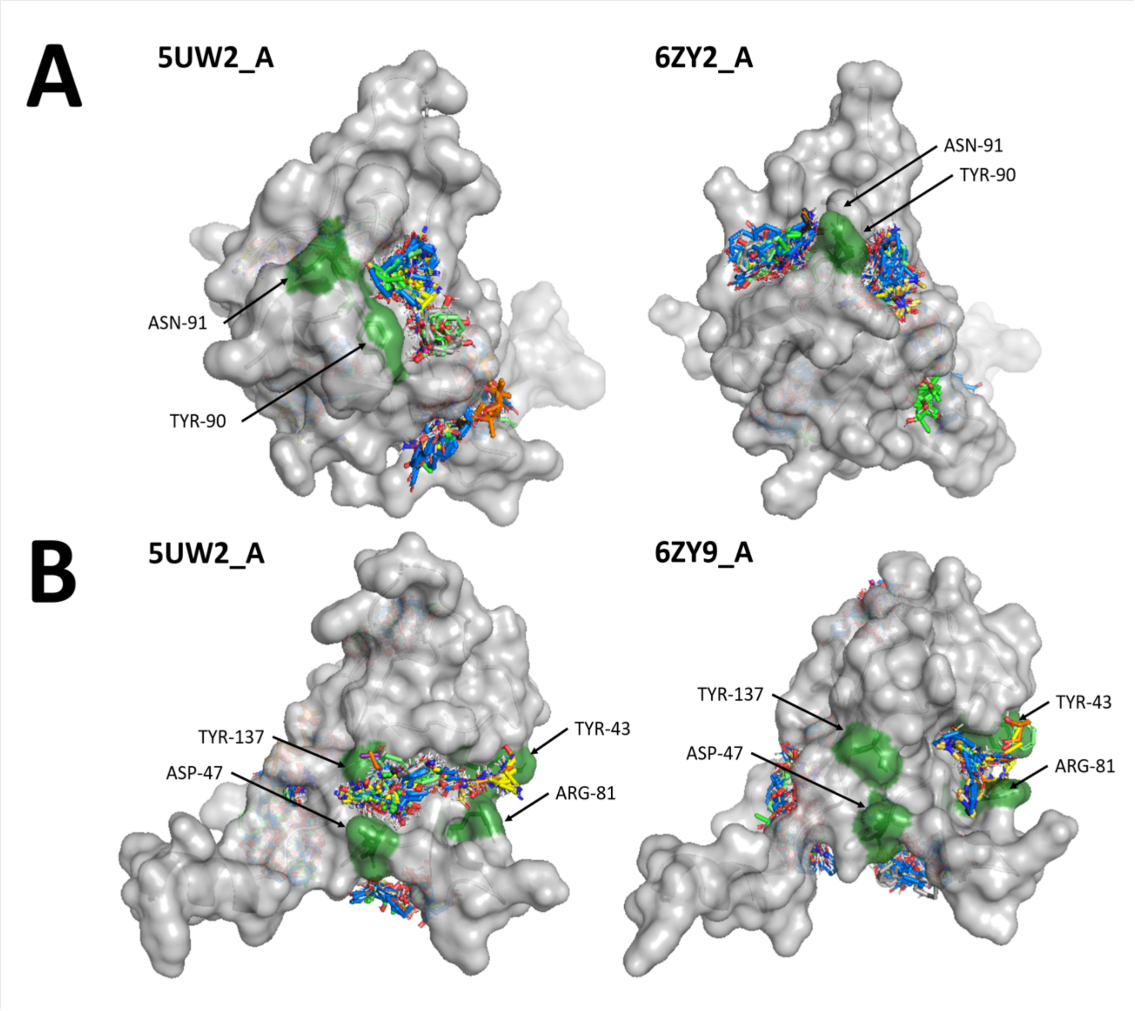

Figure 1 SI. Target T0866 side chain placement for homologous structures in the PDB with low surface property conservation. **A)** Binding fingerprint PCC between 5UW2_A and 6ZYA_A is 0.29, well below the average homolog binding fingerprint PCC. The structures appear very similar but an additional binding site is found in 6ZY2_A, relative to 5UW2_A. We propose this difference may be due to the side chain placements of TYR-90 & ASN-91 that appear to close this binding pocket in 5UW2_A, but open it up in 6ZY2_A. **B)** Binding fingerprint PCC between 5UW2_A and 6ZY9_A is 0.41, also below the average homolog binding fingerprint PCC. Again, the structures appear very similar, but small coordinated residue movement appears to close off part of a binding pocket on 6ZY9_A, relative to 5UW2_A. We propose the residues responsible for this change in binding site strength may be TYR-137, ASP-47, TYR-43, and ARG-81.
